# Benefits of public awareness in mitigating cystic echinococcosis risk in Western China: A climate and socio-economic perspective

**DOI:** 10.1371/journal.pntd.0013182

**Published:** 2025-07-09

**Authors:** Fang Yin, Wenrui Meng, Peiwei Fan, Yue Shi, Shuai Chen, Yongchun Liang, Jianyi Yao, Yeping Wang, Chuizhao Xue, Shuai Han, Mengmeng Hao, Qian Wang, Ze Meng, Jun Zhuo, Kai Sun, Yongqing Bai, Tingting Kang, Zhenyu Wang, Lei Liu, Dong Jiang, Liqun Fang, Canjun Zheng, Jiping Dong, Fangyu Ding, Tian Ma

**Affiliations:** 1 School of Land Engineering, Chang’an University, Xi’an, Shaanxi, China; 2 Institute of Geographic Sciences and Natural Resources Research, Chinese Academy of Sciences, Beijing, China; 3 College of Resources and Environment, University of Chinese Academy of Sciences, Beijing, China; 4 Chinese Center for Disease Control and Prevention, Beijing, China; 5 School of Earth Science and Resources, Chang’an University, Xi’an, Shaanxi, China; 6 National Institute of Parasitic Diseases, Chinese Center for Disease Control and Prevention, Beijing, China; 7 Nuffield Department of Medicine, Centre for Tropical Medicine and Global Health, University of Oxford, Oxford, United Kingdom; 8 Mahidol Oxford Tropical Medicine Research Unit, Faculty of Tropical Medicine, Mahidol University, Bangkok, Thailand; 9 School of Engineering, Tibet University, Lhasa, Tibet, China; 10 GeoAI Lab, Department of Geography, University at Buffalo, Buffalo, New York, United States of America; 11 State Key Laboratory of Remote Sensing Science, Aerospace Information Research Institute, Chinese Academy of Sciences, Beijing, China; 12 School of Urban Planning and Design, Peking University, Shenzhen Graduate School, Shenzhen, China; 13 School of Land Science and Technology, China University of Geosciences (Beijing), Beijing, China; 14 Academy of Military Medical Science State Key Laboratory of Pathogen and Biosecurity, Beijing Institute of Microbiology and Epidemiology, Beijing, China; 15 Yale Institute for Biospheric Studies, Yale University, New Haven, Connecticut, United States of America; 16 School of the Environment, Yale University, New Haven, Connecticut, United States of America; Consejo Nacional de Investigaciones Cientificas y Tecnicas, Fundación Mundo Sano, ARGENTINA

## Abstract

**Background:**

The prevalence of cystic echinococcosis (CE), a widespread zoonotic disease, imposes a significant public health burden, especially in western China. However, under the background of global change, how to meet the challenge of the future risk of CE remains unclear. As global climate change, land use changes, and socio-economic factors continue to progress, the spread and intensity of CE may potentially worsen, making it crucial to assess and mitigate future risks.

**Methods:**

By employing Bayesian additive regression trees model to develop risk models for CE in animal hosts (cattle, sheep and dogs) and humans, this study mapped the current distribution of infection risk for CE and projected future risks under the SSP2-4.5, SSP3-7.0, and SSP5-8.5 scenarios. The projections considered both constant and increased rates of public awareness rates regarding CE prevention in the future.

**Results:**

Current simulations indicate that the regions with a high risk of CE infection are primarily concentrated in Tibet, Qinghai, Gansu, and Xinjiang. Future projections suggest that heightened CE risks will be experienced in regions such as Yunnan, Gansu, and Sichuan will experience heightened CE risks. Notably, predictions suggest that increased public awareness is estimated to be linked to accompanied by a reduction of the population at risk by 2.72% to 3.35% in western China by 2030.

**Conclusion:**

This research offers a comprehensive understanding of the future distribution of epidemic risk for CE under climate and socio-economic changes. It highlights that enhancing public awareness regions with high-risk is a critical factor associated with reduced infection rates. Furthermore, the study offers a valuable framework for assessing the risk associated with other zoonotic diseases.

## Author summary

Cystic echinococcosis (CE) is a zoonotic disease prevalent in western China. In this study, we used a risk model to predict how the spread of CE might change in the future, considering factors like climate change, land use changes, and socio-economic development. We focused on understanding how these changes might affect high-risk areas and what role public awareness plays in reducing the spread of disease. Our results suggest that regions such as Yunnan, Gansu, and Sichuan may experience higher risks in future. Importantly, we found an association between improved public awareness of CE prevention and an estimated reduction of the population at risk by 3.35%, corresponding to a decrease of 7.92 million people in western China by 2030. This study provides valuable insights into how global changes may influence the future spread of CE and emphasizes the importance of public health interventions. Our findings offer a useful framework for assessing the risk of other zoonotic diseases that may be influenced by similar factors.

## Introduction

*Echinococcus granulosus* (*E. granulosus*), is a parasite that can cause a serious zoonotic disease called cystic echinococcosis (CE) [1]. Its adult tapeworm parasitizes carnivores and its larvae parasitizing various herbivorous livestock and humans. The distribution of CE is extensive and has expanded in parallel with the development of global livestock resources, thus emerging as a significant global public health concern. The World Health Organization (WHO) has identified it as one of the 21 neglected tropical diseases (NTDs) [2].

The life cycle of *E. granulosus* involves intermediate hosts, such as cattle and sheep, and definitive hosts, typically dogs [2]. Infected canines excrete the parasite’s eggs in their feces, thereby contaminating soil, grasslands, and water sources. Intermediate hosts acquire the infection through grazing on contaminated lands [3,4]. Humans serve as accidental hosts and can become infected by ingesting eggs through contaminated food or water or through direct contact with fecal matter from infected dog. Once ingested, the eggs hatch, migrate through the bloodstream, and lodge in organs, forming cysts that cause severe pathological damage [5].

CE leads to substantial economic losses in both the livestock industry and human healthcare [3,6–9]. In livestock, the disease causes organ damage such as the liver and lungs, reducing the quality and quantity of meat, milk, and wool production. Additionally, it hampers reproductive rates while increasing costs associated with handling infected viscera and deceased animals, and can even result in export bans on animals and their products [10,11]. For humans, the economic burden stems from medical treatments, rehabilitation efforts, as well as a decline in life quality caused by disability. In certain endemic regions worldwide such as Argentina, Peru, East Africa, Central Asia, and China, human incidence rates can exceed 50 per 100,000 person-years while prevalence rates ranging from 5% to 10% have been observed [12]. Globally, CE results in an estimated annual economic losses of USD 3 billion and accounts for approximately one million disability-adjusted life years (DALYs) [13].

China bears a significant portion of the global CE burden, with nearly 400,000 DALYs annually, representing 40% of the global total [14]. This disease burden is primarily concentrated in western provinces such as Tibet, Gansu, Qinghai, Ningxia, Xinjiang, Inner Mongolia and Sichuan. Since the 1950s, over 35,000 human CE cases have undergone surgical treatment in China. A study conducted in Ningxia in 2008 revealed that the average economic burden for CE patients undergoing surgery exceeded USD 1,500 [15,16]. In addition, it is estimated that around 30 million livestock are affected by CE in China resulting in an average economic cost of approximately USD 140 million [17–19].

The prevalence of CE is associated with environmental conditions and the distribution of animal hosts [20]. Environmental factors, such as climate and land use, can influence the transmission cycle of *E. granulosus* by affecting egg survival and the interaction between animal hosts and humans [21–24]. Several studies have demonstrated a significant correlation between CE prevalence and temperature, precipitation, elevation and landscape [21,23,25,26]. Changes in environmental conditions that facilitate the transmission between definitive and intermediate hosts may increase the risk of human CE [24,27]. Moreover, public awareness of CE prevention plays a crucial role in mitigating the risk of infection [28,29]. Lack of knowledge about preventive measures can exacerbate infection rates, highlighting the necessity for future strategies aimed at enhancing awareness [30]. In fact, a global projection based on burden of disease estimates has indicated that, without intervention, the number of CE cases could exceed 200,000 by 2030, representing a 13.63% increase from 2019 [31]. To address these challenges comprehensively, the World Health Organization has established impact-oriented global targets within the “2021-2030 Roadmap for Neglected Tropical Diseases”, which encompasses bolstering CE control efforts in 17 highly endemic countries by 2030 [32].

The implementation of a comprehensive control plan for CE is currently underway in China, with the aim of achieving a 98% awareness rate among primary and secondary school students in endemic regions by 2030 [33]. Despite these commendable efforts, there remains a dearth of studies examining the projected risk of CE in response to future global changes, as well as simulations exploring the potential impact of increased public awareness on CE transmission risk.

Therefore, in order to bridge this knowledge gap, we developed spatial risk models for CE in animal hosts (cattle, sheep, and dogs) and humans based on the 2012–2016 echinococcosis survey data in China [19]. Using Bayesian Additive Regression Trees (BART), we predicted the future distribution of CE infection risk in western China by the 2030s, while considering future climate and social-economic changes. Furthermore, we simulated potential CE risk scenarios under both unchanged and increased awareness rates. This will aid relevant government agencies in strengthening their surveillance and early warning capabilities for CE in China, providing a scientific foundation and strategies for targeted prevention and control measures in the future.

## Materials and methods

### Data collection and processing

#### Disease data.

Township-level prevalence data of CE in humans, dogs, sheep and cattle infection risk was retrieved from the National Echinococcosis Survey conducted by the Chinese Center for Disease Control and Prevention (China CDC). The survey data covers the period from 2012 to 2016. The study area primarily includes 409 counties in nine provinces in China where CE is endemic (Inner Mongolia, Gansu, Qinghai, Tibet, Sichuan, Ningxia, Xinjiang, Yunnan, and Shaanxi). In these areas, reported cases of human CE account for 98% of all reported cases in China [19]. In accordance with the established protocol, 16 villages were randomly selected from each surveyed county, and in each village, 200 residents aged over one year underwent ultrasound examinations. For those with suspected cases, blood serum was collected for further analysis, and the presence of Echinococcus antibodies was tested using ELISA. Diagnosis and classification were carried out based on the “Diagnostic Criteria of echinococcosis” of China (WS 257–2006), which is consistent with the World Health Organization guidelines [19,23].

Positive points were generated within townships with reported cases, while negative points were generated in townships without reported cases, ensuring the number of negative points matched the number of positive points. The selection of negative points followed these principles: we established a buffer zone with a radius of 30 km around the center of each township administrative division and conducted an overlay analysis to remove overlapping negative points, ensuring only one negative point remained within each 30 km x 30 km grid, which helped to eliminate biases that may arise from cluster effects during the modeling process [34], code written in R to complete this process. These points serve as the dependent variables for township-level modeling to analyze risk probabilities, which include the predicted infection risk probabilities for cattle, sheep, and dogs.

#### Spatial influence factors.

Based on previous studies of CE, a set of ecoclimatic, geographic, socio-economic, and biological factors that could potentially influence the prevalence of CE were identified and incorporated as explanatory covariates for risk area simulation in this study (S1 Table). These variables were obtained from different data repositories, with detailed information on spatial resolution, study duration, and sources provided in S2 Table. In this study, average values for each township during the period between 2007 and 2016 were computed for ecoclimatic conditions, land cover, population density, and awareness rates. The awareness rate at the county level was obtained from a questionnaire survey conducted by China CDC. This survey primarily explored infection routes, hygiene practices, interactions with dogs, handling of livestock post-slaughter, and attitudes towards dog deworming initiatives, and their awareness was rated on a scale from 0 to 100, with a score of 60 indicating adequate awareness [23]. For counties with missing data, interpolation was performed using the average values from neighboring counties for areas of the study region that were not surveyed, resulting in the per capita awareness rate of CE at the county level across the study area. This variable reflects an ecological-level estimate of public awareness and is not linked to individual-level infection outcomes. Additionally, elevation data from 2010 and cattle and sheep density data from 2015 were included in our analysis.

To model the current and future risk distribution of CE, two time periods were selected: (i) 2007–2016, focusing on historical risk areas for animal hosts and human cases using average values of ecoclimatic conditions, land cover, and population density. Elevation data from 2010 was included along with cattle and sheep densities from 2015 as fixed independent variables. (ii) 2020–2040, for predicting future risk distributions, we used ecoclimatic factors derived from four General Circulation Models (GCM models)-ACCESS-CM2, BCC-CSM2-MR, CMCC-ESM2, and EC-Earth3-Veg under the SSP2-4.5, SSP3-7.0, and SSP5-8.5 scenarios respectively. Furthermore, projected data for land cover and population density under the same future scenarios were used as sources of future covariate data, while assuming constant values for elevation as well as cattle and sheep densities due to a lack of future predictions.

### Modeling methods

#### Analytical models.

The Bayesian additive regression trees (BART) is used in this study for species distribution modeling, and subsequent model predictions are made using the dbarts package implemented in the R package embarcadero [35].

Compared to other classification and regression tree (CART) methods, BART is an integrated method that combine Bayesian theory with the cumulative tree model, which applies prior information to the parameters of the cumulative tree model [36,37]. In the single tree model (single binary regression tree), tree *T* is a binary tree consisting of a set of internal node decision rules and a set of leaf nodes. *M*={*μ*_*1*_*, μ*_*2*_, ……*, μ*_*b*_} represents a set of parameter values associated with all *b* leaf child nodes of *T*. Specifically, the segmentation rule of non-leaf nodes is a binary segmentation of the value domain space of the input variables, in the form of {*x *∈* A*} and {*x *∉ *A*}, where *A* is a subset of the input variable. Each is made from top to bottom to be associated with a leaf node of tree *T*, and its associated parameter *μ*_*i*_ is the response value of the input x. Given *T* and *M*, the model of a single tree can be expressed as:


$$Y=g(x;T,M)+\varepsilon ,\;\varepsilon \sim N\left(0,\sigma^{2}\right)$$ (1)

Where *g* (*x; T, M*) is a function of mapping {*μ*_*i *_∈* M*}, *ε* is the Gaussian noise of the number of samples, and *ε* follows *N* (*0,* σ^*2*^). Thus, the additive tree model can be expressed as:


$$\begin{array}{c} Y=\sum_{j-1}^{m}g\left(x;T_{j},M_{j}\right)+\varepsilon ,\;\varepsilon \sim N\left(0,\sigma^{2}\right) \end{array}$$ (2)

Where *T*_*j*_ represents each binary regression tree, *M*_*j*_ represents the parameter set of leaf nodes associated with *T*_*j*_, x is mapped to the {*μ*_*ij *_∈* M*} by the function *g (x; T*_*j*_*, M*_*j*_). When the number of trees, m, is greater than 1, each *μ*_*ij*_ merely represents a fraction of the final predicted value. When *g* (*x; T*_*j*_*, M*_*j*_) has only one variable *x*, each *μ*_*ij*_ merely represents the main effect result of this variable. When g (*x; T*_*j*_*, M*_*j*_) depends on multiple variables *x*, *μ*_*ij*_ is expressed as the result of the interaction of multiple variables *x* [38].

Since the parameters (*T*_*1*_*, M*_*1*_), (*T*_*2*_*, M*_*2*_), ……, (*T*_*m*_*, M*_*m*_) and *ε* determine the BART model, prior information needs to be added to those parameters. For a given sample dataset, the joint posterior distribution of the model parameters can be obtained by applying a Bayesian approach and then using sampling to obtain the parameter values. BART model parameter training uses Bayesian Backfitting MCMC (Markov Chain Monte Carlo) algorithm, which is a combination of Bayesian Backfitting fitting strategy and MCMC repeated sampling algorithm.

The BART model mentioned above is based on continuous response variables, while the BART model can also use logit link to deal with binary classification problems, whose formula is:


$$\begin{array}{c} Y=\Phi [\sum_{j-1}^{m}g\left(x;T_{j},M_{j}\right)] \end{array}$$ (3)

where Φ is the cumulative distribution function of the standard normal distribution. [39]

#### Modeling and future risk predictions.

To mitigate the impact of high correlation among the 19 bioclimatic variables on the accuracy of prediction results, we initially performed Principal Component Analysis (PCA) to assess the correlation structure and reduce redundancy among variables. Based on the variable loadings in the principal components and the cumulative variance explained (99%), we selected nine representative variables: BIO2, BIO6, BIO8, BIO9, BIO12, BIO13, BIO14, BIO15, and BIO19. These variables capture the major environmental gradients while retaining ecological interpretability.

We further conducted a correlation analysis to identify variable pairs with a correlation coefficient surpassing 0.8, with the results of the analysis displayed in S1 and S2 Figs. Subsequently, we conducted Variance Inflation Factor (VIF) analysis, with the results presented in S3 Table. Specifically, variables exhibiting a VIF value exceeding 10 or a correlation coefficient surpassing 0.8 were identified as having multicollinearity issues and subsequently excluded [40,41]. Then, the BART model was employed to predict the risk of CE in three main animal hosts (cattle, sheep, and dogs) at the township level. Each model utilized a distinct combination of variables selected based on VIF analysis and correlation analysis (S4 Table). Subsequently, using selected ecoclimatic, geographical, and socio-economic variables, we first estimated the infection risk for dogs, cattle, and sheep. Then, by incorporating these predicted animal hosts infection risks into the human CE modeling analysis, we predicted the risk of human infection with CE. The previously processed data on human CE positive and negative points were used as independent variables in the township-level modeling analysis. Additionally, in the human CE modeling process, two strategies were considered: one including awareness rate and another excluding it.

The data from each animal host and human cases was randomly split into an 80% training set and a 20% test set. The method of 10-fold cross-validation was used to fit 100 models by repeating the process 100 times. The final estimation results and overall predictive performance are represented by the average values of the Relative Contributions (RC) of all predictors, the Area Under the Receiver Operating Characteristic Curve (AUC), Root Mean Square Error (RMSE), and F1-score, which were calculated across 100 models on the test sets. RMSE serves as a measure of the average magnitude of the errors, with lower values indicating a better fit between the predicted and actual values. The F1-score acts as a balanced measure of a model’s accuracy, combining precision and recall, especially useful in situations with imbalanced classes. Lastly, the standard deviation was calculated based on 100 predictions to represent the model uncertainty.

The assessment of variable importance in BART models is typically measured by calculating the number of times a given variable is used in the complete posterior probability sampling of the trees. Variable response curves are generated by applying the BART model to predict each variable over its range of values. The standard deviation of variable importance and the confidence intervals of variable response curves are calculated through 100 repeated runs of the model. Besides, the R package embarcadero includes a special tool that can draw spatial partial dependence plots and reclassify prediction raster based on partial dependence plots of predictor variables, displaying the relative suitability of different areas for a single covariate [35].

To further assess the impact of future change on CE risk, we utilized projected climate data from four GCM models under three scenarios to predict the risk of CE infection in animal hosts and humans in the 2030s based on the BART model. By averaging the future prediction results derived from the four GCM models, we obtained more robust predictions. This process can provide a comprehensive evaluation of future risk distributions.

## Results

### Analysis of driving factors for CE

The ensembled BART models for predicting the presence of CE in animal hosts and humans demonstrated good performance (Table 1). The human CE risk model, which incorporated the awareness rate, showed improved performance compared to the model excluding awareness, with higher AUC and F1 scores and lower RMSE values. As a result, subsequent analyses focused on the results of the model incorporating the awareness rate.

**Table 1:** Model accuracy for predicting CE infection risk.

Infection Risk Model	AUC	RMSE	F1 Score
**Cattle Infection Risk Model**	0·80	0·44	0·67
**Sheep Infection Risk Model**	0.78	0.44	0.69
**Dog Infection Risk Model**	0.73	0.46	0.67
**Human CE Risk Model (Excluding Awareness)**	0.76	0.44	0.69
**Human CE Risk Model (Including Awareness)**	0.81	0.42	0.72

**Fig 1:**
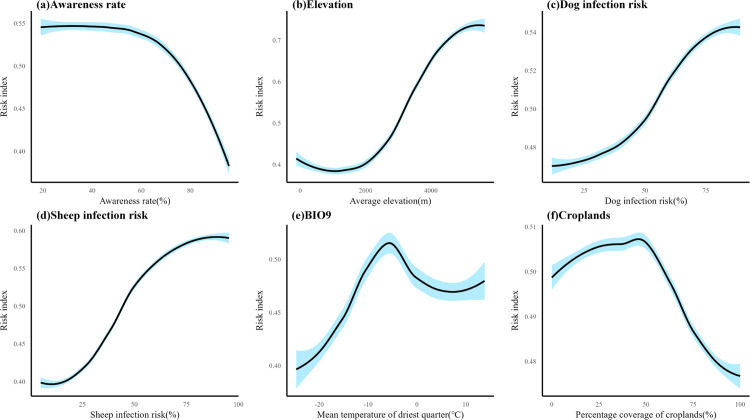
Response curve for the six most important risk factors in the human CE risk model (including awareness rate). The blue shading gives a 95% credible interval from the posterior distribution of the Bayesian model.

Predictive models for animal hosts were constructed based on selected variables, with the importance of each variable illustrated in S3 Fig. S4-S6 Figs provide the response curves for the top six variables influencing the infection risk in each animal host. In the human CE risk model, the awareness rate emerged as the most influencing factor (mean importance: 7.52% ± 0.15%), followed by elevation (mean importance: 7.46% ± 0.17%), and the infection risks of dogs and sheep. Fig 1 presents the response curves for the top six variables in the human CE risk model. As shown, awareness rate has a negative effect on human CE risk, while elevation, dog infection risk, and sheep infection risk are positively correlated with human CE. In addition, these risk models also exhibited low uncertainty levels as shown in S7 Fig.

**Fig 2:**
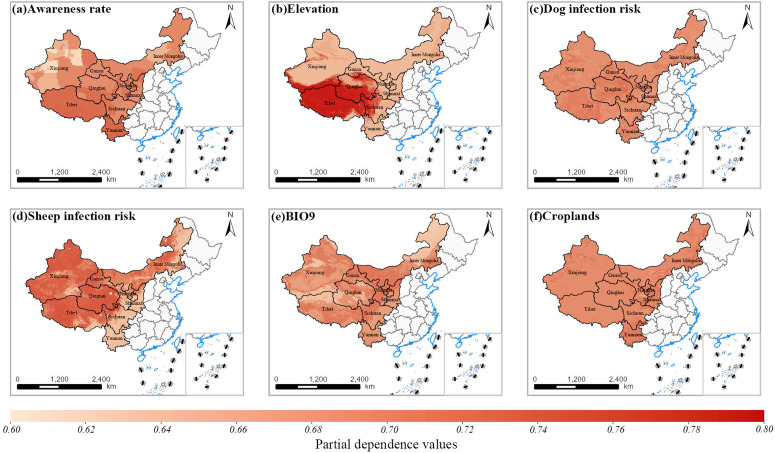
Spatial partial dependence plots in human CE risk model. Variables are given in descending order of the first six places of model importance. Values are unitless partial effects on predicted probabilities that range from 0.6 to 0.8. Note: Base map data from Map World (https://map.tianditu.gov.cn/).

The spatial partial dependency plots of the six most important variables for the animal infection models were generated to visualize the effect of individual covariates across different geographic areas (S8-S10 Figs). Fig 2 presents the spatial partial dependence plots for the six most important variables related to human CE risk. The plots show that higher-elevation areas, particularly on the Qinghai-Tibet Plateau, exhibit increased disease risk, and regions with higher infection risks in sheep and dogs correspond to higher human CE risk. In contrast, areas with higher awareness rates demonstrate significantly reduced CE risk.

### Current distribution of CE infection risk

**Fig 3:**
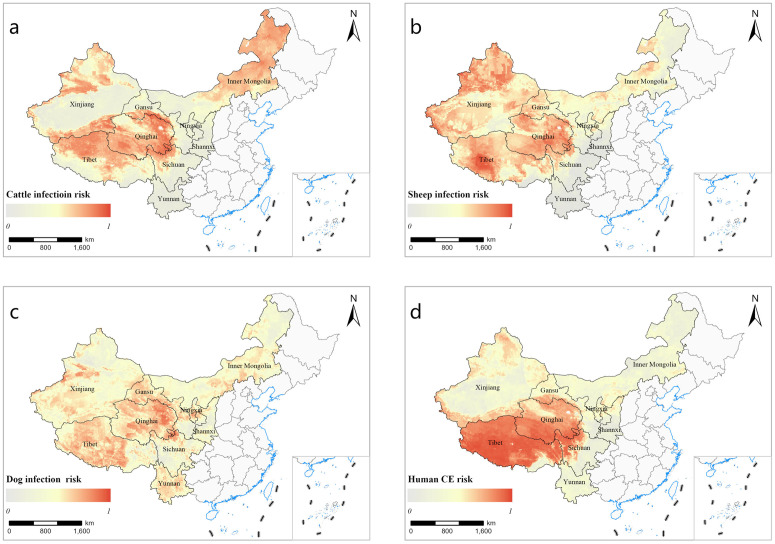
The current spatial distribution of predicted CE infection risk based on the modelling analysis. (a)cattle, (b)sheep, (c)dog, (d)human CE. Note: Base map data from Map World (https://map.tianditu.gov.cn/).

Fig 3 illustrates the current spatial distribution of predicted CE infection risk for cattle, sheep, dogs, and humans. The analysis shows that high CE infection risk for animal hosts is predominantly concentrated in Qinghai, Gansu, and Xinjiang. Dogs exhibit high-risk areas extending into Yunnan, while cattle infection risk is mainly found in Qinghai, northern Sichuan, Tibet, and northeastern Inner Mongolia. Similarly, human CE infections are most prevalent in Tibet, Qinghai, Xinjiang, and northern Sichuan.

To quantify the extent of risk, a threshold of 0.5 was established to define high-risk and low-risk areas for human CE infection. The population proportion in potential high-risk areas relative to the total population in each region was calculated (Table 2). For example, Tibet and Qinghai exhibit the highest proportions of their populations residing in potential high-risk areas, with 75.93% and 46.09%, respectively. Other regions such as Xinjiang, Gansu, and Ningxia also show significant population proportions residing in potential high-risk areas (>5% of all populations).

**Table 2:** The population proportion in the provinces of western China residing in potential high-risk areas.

Province	Populations proportion at potential high-risk areas (%)	Populations number at potential high-risk areas (Million)
**Tibet**	75.93	3.08
**Qinghai**	46.09	2.66
**Xinjiang**	14.95	3.35
**Ningxia**	8.71	0.56
**Gansu**	7.01	1.80
**Inner Mongolia**	4.9	1.24
**Shaanxi**	3.81	1.43
**Sichuan**	1.68	1.35
**Yunnan**	0.47	0.22
**Total**	6.18	15.69

### Future distribution of human CE risk with unchanged and increased awareness rates

The predicted risk of CE among cattle, sheep, and dogs under three future scenarios (SSP2-4.5, SSP3-7.0, and SSP5-8.5) is shown in S11 Fig. Comparisons between the future predictions with current infection risk are presented in S12 Fig. Additionally, the future predictions for both animal hosts’ and humans’ future predictions exhibit low uncertainty, as shown in S13 and S14 Figs, respectively.

**Fig 4:**
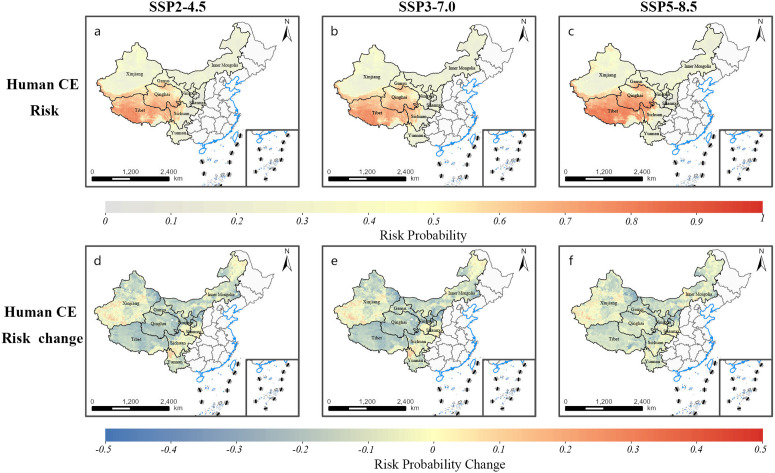
Future predicted infection risk distribution and changes of human CE in western China based on the modelling analysis. (Strategy B: Awareness Rate Increased). Note: Base map data from Map World (https://map.tianditu.gov.cn/).

The future human CE risk predictions were conducted using two strategies: Strategy A, which assumed a constant awareness rate, and Strategy B, which assumed an increased awareness rate of 90%. We compared the results of these strategies to evaluate the impact of enhanced awareness on future CE risk. Figs 4 and S15 illustrate the future spatial distribution of CE infection risk under both strategies. The population proportion and size residing in potential high-risk areas in various western provinces by the 2030s were quantified and compared to those in the 2010s (S5 and S6 Tables). Table 3 highlights the changes, suggesting that with increased awareness, the population in potential high-risk areas may decrease by 6.99 to 7.92 million individuals, accompanied by a decrease in the proportion of the at-risk population by 2.72% to 3.35% compared to the scenario of constant awareness. This substantial reduction was particularly evident in provinces such as Qinghai, Xinjiang, and Gansu, where the enhancement of local awareness coincided with a decrease in populations residing in potential high-risk areas. Specifically, in Qinghai, the high-risk population decreased by 0.54 to 1.11 million individuals, with a reduction in proportion from 11.85% to 22.28%; in Xinjiang, it decreased by 2.04 to 2.19 million individuals, with a reduction in proportion from 10.38% to 11.93%; and in Gansu, it declined by 1.67 to 2.39 million individuals, with a reduction in proportion from 3.27% to 9.63%.

**Table 3:** Changes in population proportion (%) and population size (Million) residing in high-risk areas with increased awareness rate (Strategy B - Strategy A).

Province	Changes in population proportion residing in potential high-risk areas			Changes in population size residing in potential high-risk areas		
	SSP2-4.5 (%)	SSP3-7.0 (%)	SSP5-8.5 (%)	SSP2-4.5 (Million)	SSP3-7.0 (Million)	SSP5-8.5 (Million)
**Qinghai**	–21.83	–22.28	–11.85	–1.04	–1.11	–0.54
**Xinjiang**	–10.72	–10.38	–11.93	–2.04	–2.04	–2.19
**Gansu**	–8.19	–6.27	–9.63	–2.11	–1.67	–2.39
**Ningxia**	–2.8	–0.95	–12.8	–0.17	–0.06	–0.75
**Tibet**	–2.77	–2.71	–2.46	–0.08	–0.08	–0.06
**Yunnan**	–3.47	–3.48	–0.48	–1.37	–1.45	–0.18
**Inner Mongolia**	–1.62	–0.62(–0.17)	–4.44	–0.43	–0.17	–1.13
**Shaanxi**	–0.39	–0.06	–1.29	–0.15	–0.02	–0.48
**Sichuan**	–0.38(–0.32)	–0.42(–0.37)	–0.26	–0.32	–0.37	–0.21
**Total**	–3.12	–2.72	–3.35	–7.71	–6.99	–7.92

The data suggest a correlation between increased awareness rate and a reduction in the overall number of at-risk individuals, with a particularly strong association observed in provinces with larger high-risk populations, where both the number and the proportion of populations at risk tend to decrease.

## Discussion

The zoonotic infection of CE has had a significant global impact on public health, with China being the country with the highest worldwide prevalence [42]. In 2016, the Chinese government released the “Healthy China 2030” blueprint, with the objective of strengthening the prevention and control against major infectious diseases [43]. To implement this blueprint, China CDC issued a comprehensive prevention and control plan for combating key parasitic diseases in 2024, which included specific requirements regarding the awareness rate of CE related knowledge among primary and secondary school students, to effectively control the spread of CE across all endemic counties nationwide [33]. Thus, understanding the changes in the risk of CE prevalence in the context of global changes serves as a fundamental prerequisite, while understanding the impact of increased awareness on the risk of CE is key for achieving precise disease control.

Our results indicate that climatic conditions, including temperature and precipitation, significantly influence the transmission risk among various hosts (Figs S6–S8 and 1). This could be attributed to the vulnerability of *E. granulosus* transmission to climatic factors, as variations in temperature and humidity can affect the survival and suitability of eggs in the external environment, thereby impacting disease transmission [27,44,45]. Notably, a previous study found no clear relationship between temperature and CE risk in Chile, suggesting that the influence of climate factors on CE prevalence may vary across regions [46]. In addition, it is evident that the risk of CE is positively correlated with grassland and forest (S3 Fig). This correlation is likely due to the role of these landscapes as natural habitats for intermediate hosts such as cattle and sheep. These habitats provide favorable conditions for the survival of both host and parasite survival, facilitating disease transmission [25,47].

In addition to considering various environmental factors, we also included biological factors, primarily the risk of infection in the three main animal hosts for CE, into our human CE risk model. Our analysis demonstrated a nonlinear positive correlation between infection rates in animal hosts and the risk of human CE. Intermediate hosts such as sheep and cattle primarily acquire infections through ingesting feed and water contaminated with eggs from dog feces [5]. Subsequently, when definitive hosts like dogs consume infected sheep or cattle, they become infected and excrete parasite eggs into the environment, thereby increasing the contamination level and increasing the risk of human infection [48].

A key finding of this study is the notable association between public awareness and the reduction of human CE risk. Our models show that awareness is the most significant factor influencing human infection risk. Notably, increased public health awareness is linked to a marked decrease in human infection risk. This finding suggests that enhanced public education, particularly in high-risk areas, is essential for disease prevention. As the awareness assessment in this study such as knowledge of transmission routes, hygiene practices, interactions with dogs, post-slaughter livestock handling, and attitudes toward dog deworming, improvements in these areas of awareness are associated with a lower risk of human CE, suggesting the potential value of targeted public health education in disease prevention. Previous research has also demonstrated that educational campaigns can significantly reduce CE prevalence, thereby reinforcing the importance of integrating public health education into disease control strategies [30].

The future risk predictions presented in this study emphasize the potential role of increased awareness in mitigating the burden of CE in western China. Under Strategy B, when public awareness increases to 90%, the modeled risk can decrease by 4.12% compared to the 2010s baseline. On the other hand, relative to Strategy A, which assumes no change in awareness, the reduction in population at-risk might be up to 3.35%. This reduction is particularly pronounced in high-risk regions such as Tibet, Qinghai, and Xinjiang, where the population proportion residing in high-risk areas could drop by as much as 22.28% in comparison to Strategy A. Conversely, without significant improvements in public awareness, it is possible that regions such as Xinjiang, Gansu, and Yunnan are projected to experience increasing CE risk due to the lack of sufficient awareness, which leads to the continued spread of infection among animal hosts. This could result in an increase of as much as 8.43% in CE risk, while Strategy B is expected to minimize this risk by 11.93% in contrast to Strategy A.

These results emphasize the potential contribution of public health awareness to the prevention and control of CE. By improving public awareness of CE, the proportion of the population residing in areas identified as high risk is projected to decline, thereby contributing to a reduction in overall community CE risk. Additionally, the findings also highlight the importance of integrating public health education into disease control strategies. Awareness interventions that focus on key behavioral pathways such as reducing close contact with dogs, adopting hygienic practices after livestock slaughter, and promoting regular dog deworming have the potential to produce measurable reductions in infection risk. When public health awareness is increased, host animals management may also be more standardized [30], which in turn could help limit zoonotic transmission of CE and reduce future risks through preventive measures. In terms of implementation, such interventions are supported by existing government efforts. For instance, national plans under the “Healthy China 2030” initiative have explicitly promoted CE awareness among primary and secondary school students in endemic counties. This highlights the feasibility of integrating educational campaigns into existing infrastructure [33,43]. To ensure sustained impact, however, factors such as accessibility, community engagement, and cost-effectiveness, especially in rural and remote areas, must also be considered. Further research is needed to evaluate the practical implementation and long-term policy relevance of these interventions.

It is important to note that the public awareness data used in this study come from cross-sectional surveys and are not linked to individual infection status. As such, the associations found reflect ecological relationships rather than causality. While the projections suggest possible benefits of increased awareness under certain scenarios, more evidence from longitudinal or interventional studies is needed to confirm its actual impact on CE risk.

While this study provides valuable insights into the present and projected risk of CE in western China, several limitations should be considered. First, the study does not account for the distribution of stray dogs, which are key hosts for *E. granulosus*, while our study acknowledges the exclusion of stray dogs as a limitation, future work should explore targeted interventions such as stray dog population monitoring and control to better capture the full transmission dynamics. Incorporating such data in future studies could provide a more comprehensive assessment of CE transmission risk. Additionally, this study focused primarily on western China, where most CE cases are reported. Expanding the analysis to include eastern regions and other areas with emerging CE cases would enhance the generalizability of the findings. Moreover, while the importance of public awareness interventions is emphasized, future studies should further evaluate their practical implementation in rural and resource-limited settings.

## Conclusion

This study evaluated the impact of various risk factors on CE infection in animal hosts and humans in western China, integrating echinococcosis survey data with geographical, ecoclimatic, socio-economic, and biological variables. By applying Bayesian additive regression tree (BART) models, we predicted current CE infection risk for three animal hosts and humans, and further projected future risk distributions under the SSP2-4.5, SSP3-7.0, and SSP5-8.5 scenarios. The projections considered two strategies that accounted for both constant and increased public awareness regarding CE prevention. Notably, under higher awareness conditions, the proportion of the population residing in potential high-risk areas could decrease by approximately 2.72% to 3.35% by 2030, highlighting the potential of public education in mitigating infection risk. These findings highlight the critical role of incorporating awareness-related interventions into CE control strategies. Additionally, this research offers a valuable framework for assessing other zoonotic diseases and emphasizes the need for adaptive management strategies as climate and socio-economic conditions change.

## Supporting information

S1 Fig Correlation matrix of predictor variables used in the animal infection risk model. Note: Bold values indicate strong correlations (|r| > 0.8).
(TIF)

S2 Fig Correlation matrix of predictor variables used in the human infection risk model. Note: Bold values indicate strong correlations (|r| > 0.8).
(TIF)

S3 Fig Importance of variables for each model. (a) cattle, (b) sheep, (c) dogs, and (d) human CE (excluding awareness rate), (e) human CE (including awareness rate).
(TIF)

S4 Fig Response curve of risk factors for cattle infection risk model. (TIF)

S5 Fig Response curve of risk factors for sheep infection risk model. (TIF)

S6 Fig Response curve of risk factors for dog infection risk model. (TIF)

S7 Fig Model uncertainty in predicting the current risk distribution. (a) cattle, (b) sheep, (c) dogs, and (d) human CE.  Note: Base map data from Map World (https://map.tianditu.gov.cn/).
(TIF)

S8 Fig Spatial partial dependence plots for the cattle infection risk model. Note: Base map data from Map World (https://map.tianditu.gov.cn/).
(TIF)

S9 Fig Spatial partial dependence plots for the sheep infection risk model. Note: Base map data from Map World (https://map.tianditu.gov.cn/).
(TIF)

S10 Fig Spatial partial dependence plots for the dog infection risk model. Note: Base map data from Map World (https://map.tianditu.gov.cn/).
(TIF)

S11 Fig Future predicted infection risk distribution of animal hosts CE in western China based on the modeling analysis. Note: Base map data from Map World (https://map.tianditu.gov.cn/).
(TIF)

S12 Fig Future predicted changes in the infection risk distribution of animal hosts CE in western China based on the modeling analysis. Note: Base map data from Map World (https://map.tianditu.gov.cn/).
(TIF)

S13 Fig Model uncertainty in predicting the future risk distribution of animal hosts CE. (a) cattle, (b) sheep, (c) dogs. Note: Base map data from Map World (https://map.tianditu.gov.cn/).
(TIF)

S14 Fig Model uncertainty in predicting the future risk distribution of human CE. (a) Strategy A, (b) Strategy B. Note: Base map data from Map World (https://map.tianditu.gov.cn/).
(TIF)

S15 Fig Future predicted infection risk distribution and changes of human CE in western China based on the modelling analysis. (Strategy A: Awareness Rate Unchanged). Note: Base map data from Map World (https://map.tianditu.gov.cn/).
(TIF)

S1 Table Factors potentially associated with the echinococcosis used in the analysis. (DOCX)

S2 Table The spatial resolution, study duration, and source of the included data. (DOCX)

S3 Table VIF assessment for variables in different models. (DOCX)

S4 Table Detail information of variables used for modelling in CE infection risk. (DOCX)

S5 Table Predicted future population proportion and changes in the provinces of western China residing in high-risk areas compared to the 2010s (%). (DOCX)

S6 Table Predicted population size changes in the provinces of western China residing in high-risk areas compared to the 2010s (Million). (DOCX)
