# Supplementary figures and images for: Benefits of public awareness in mitigating cystic echinococcosis risk in Western China: A climate and socio-economic perspective
Source: PLoS Negl Trop Dis. 2025 Jul 9;19(7):e0013182. doi: 10.1371/journal.pntd.0013182 (PMC12240338; doi:10.1371/journal.pntd.0013182)

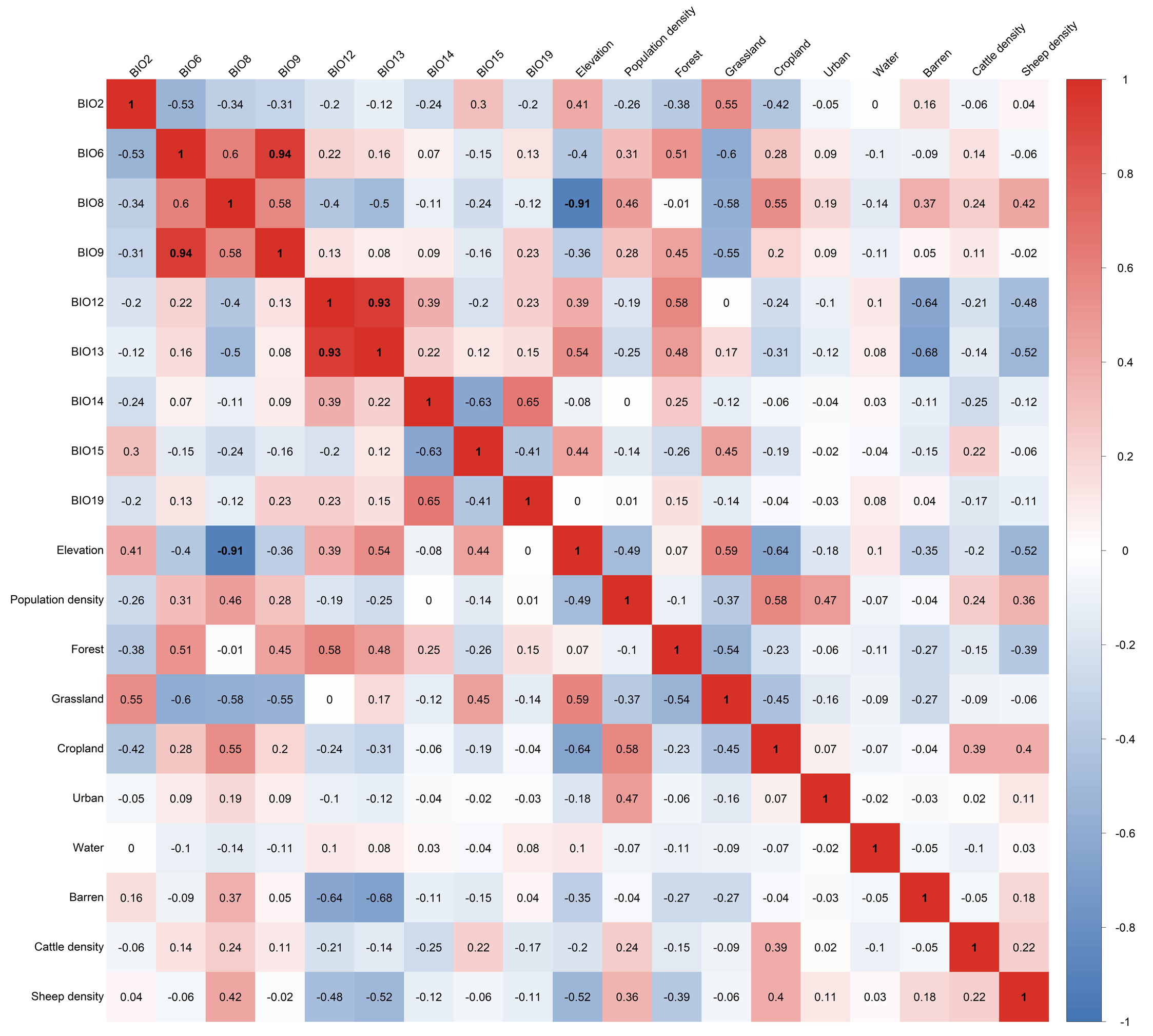

Supplement: S1 Fig — Note: Bold values indicate strong correlations (|r| > 0.8). (TIF) [file pntd.0013182.s001.tif]

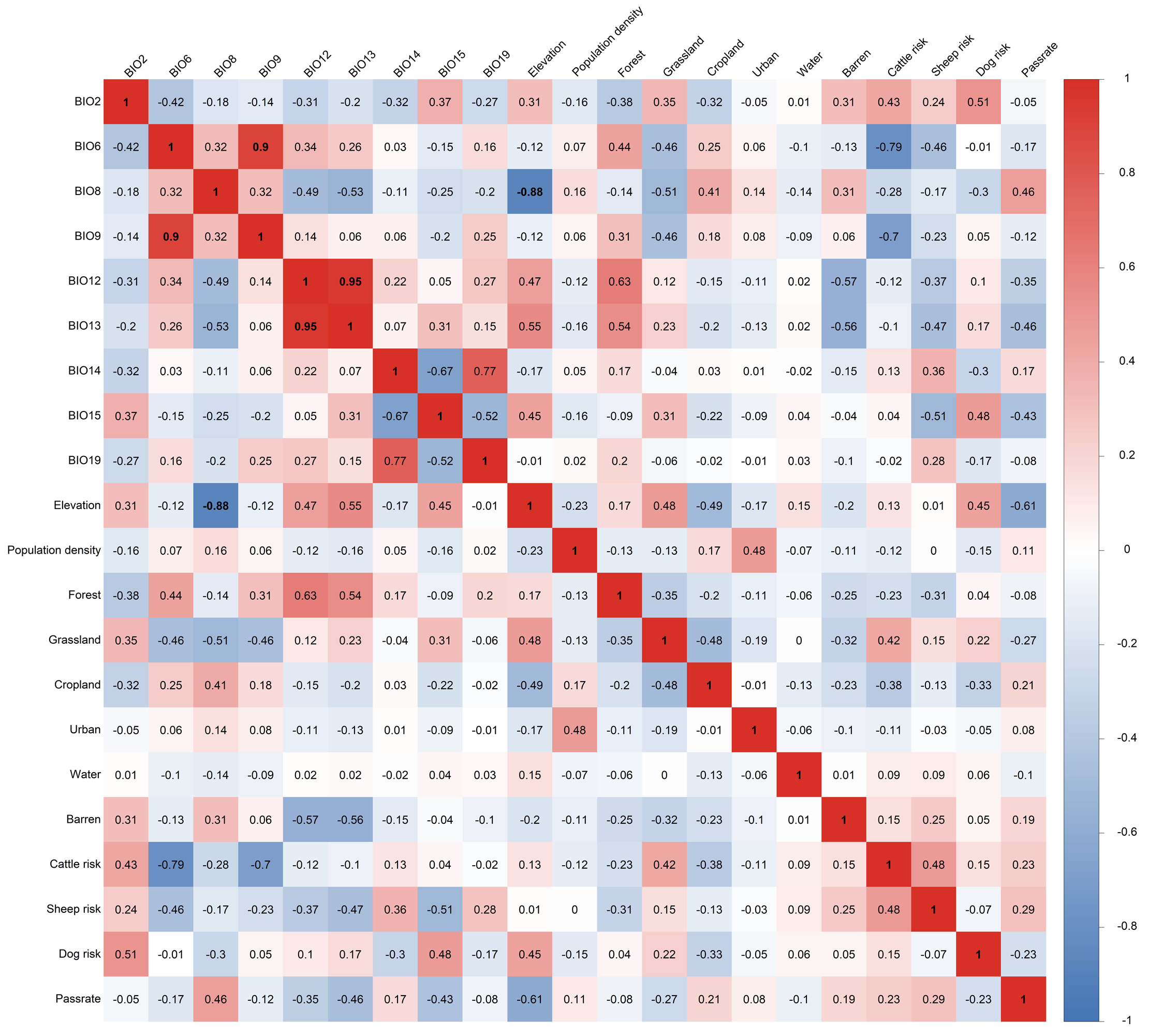

Supplement: S2 Fig — Note: Bold values indicate strong correlations (|r| > 0.8). (TIF) [file pntd.0013182.s002.tif]

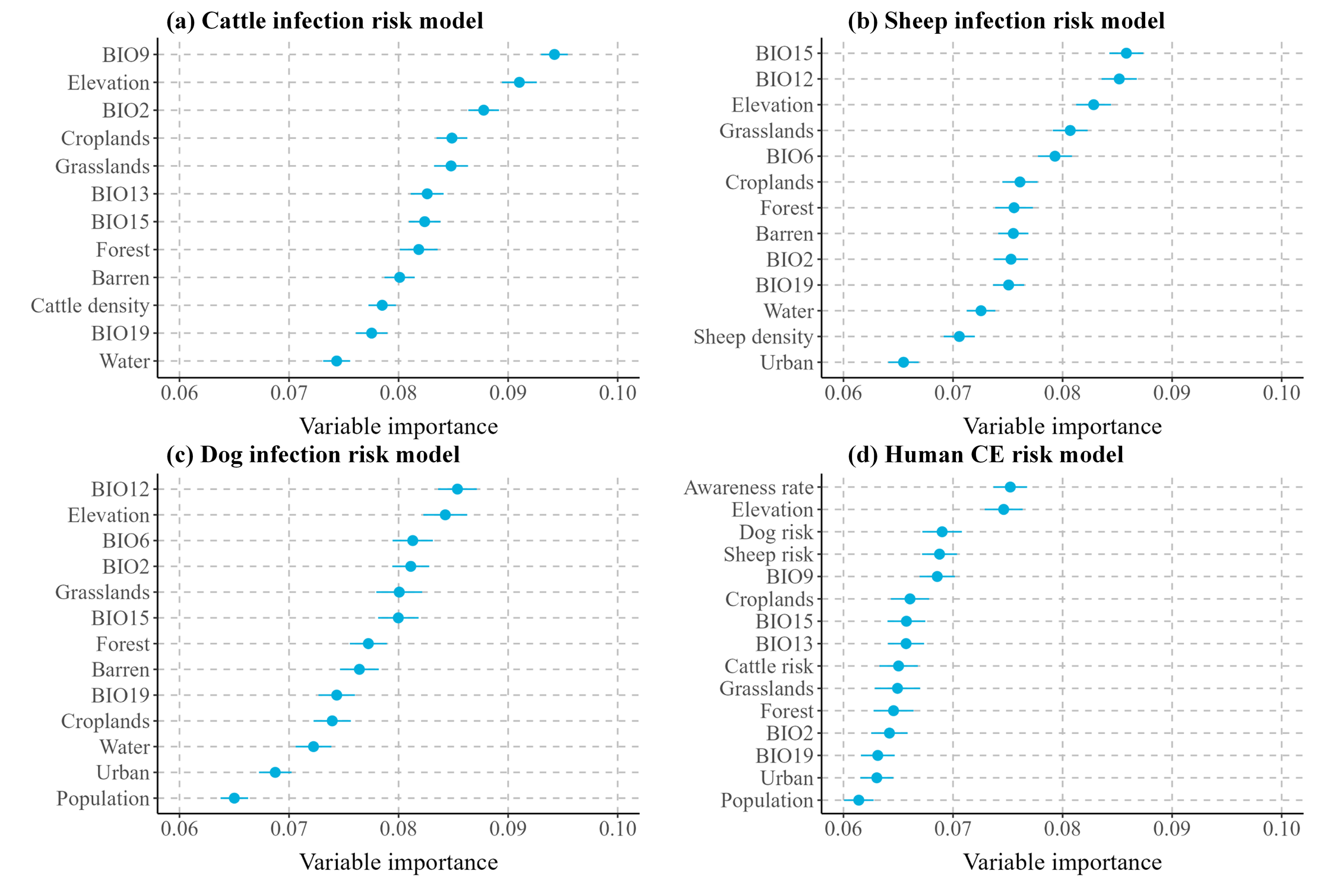

Supplement: S3 Fig — (a) cattle, (b) sheep, (c) dogs, and (d) human CE (excluding awareness rate), (e) human CE (including awareness rate). (TIF) [file pntd.0013182.s003.tif]

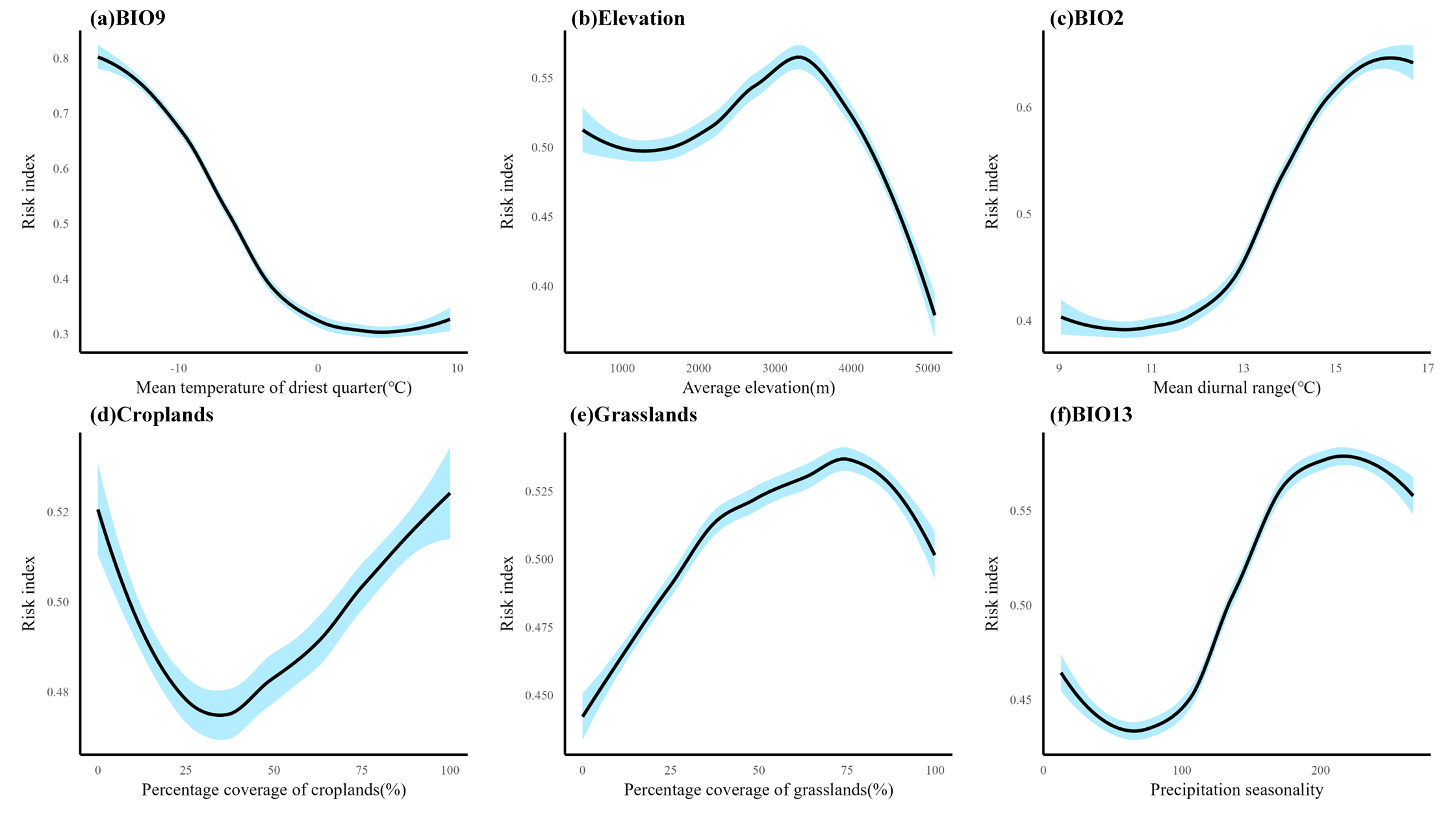

Supplement: S4 Fig — (TIF) [file pntd.0013182.s004.tif]

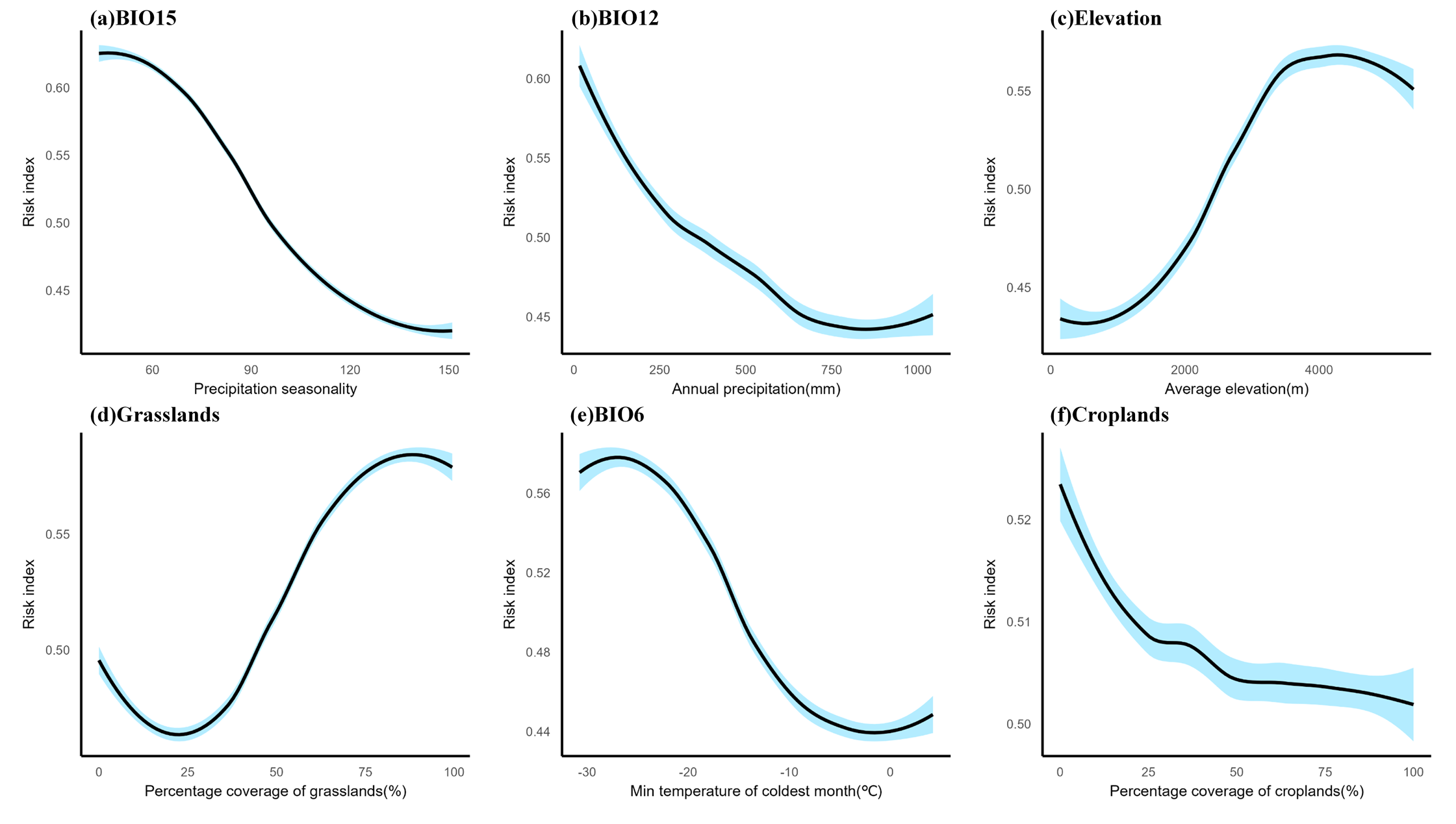

Supplement: S5 Fig — (TIF) [file pntd.0013182.s005.tif]

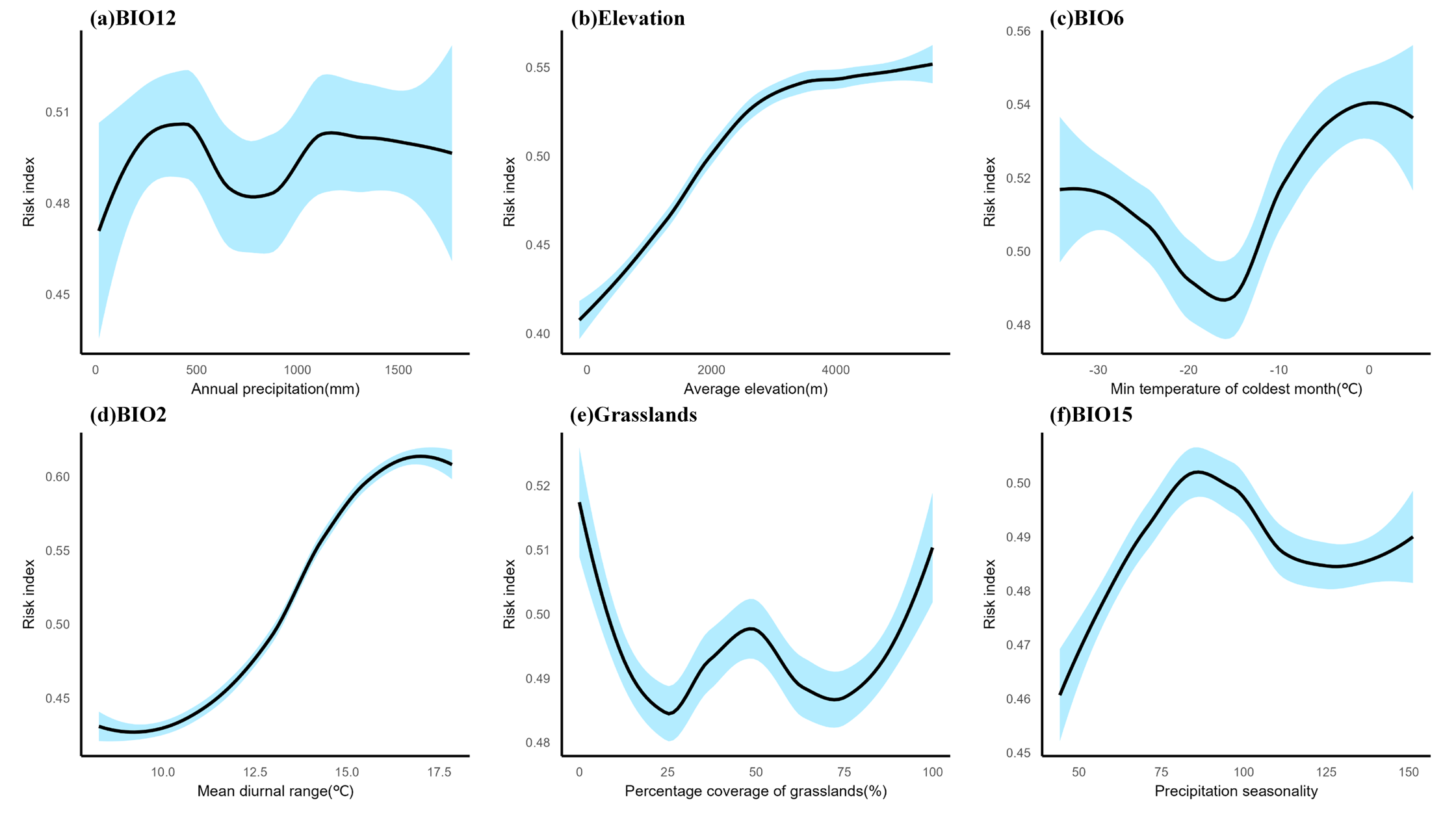

Supplement: S6 Fig — (TIF) [file pntd.0013182.s006.tif]

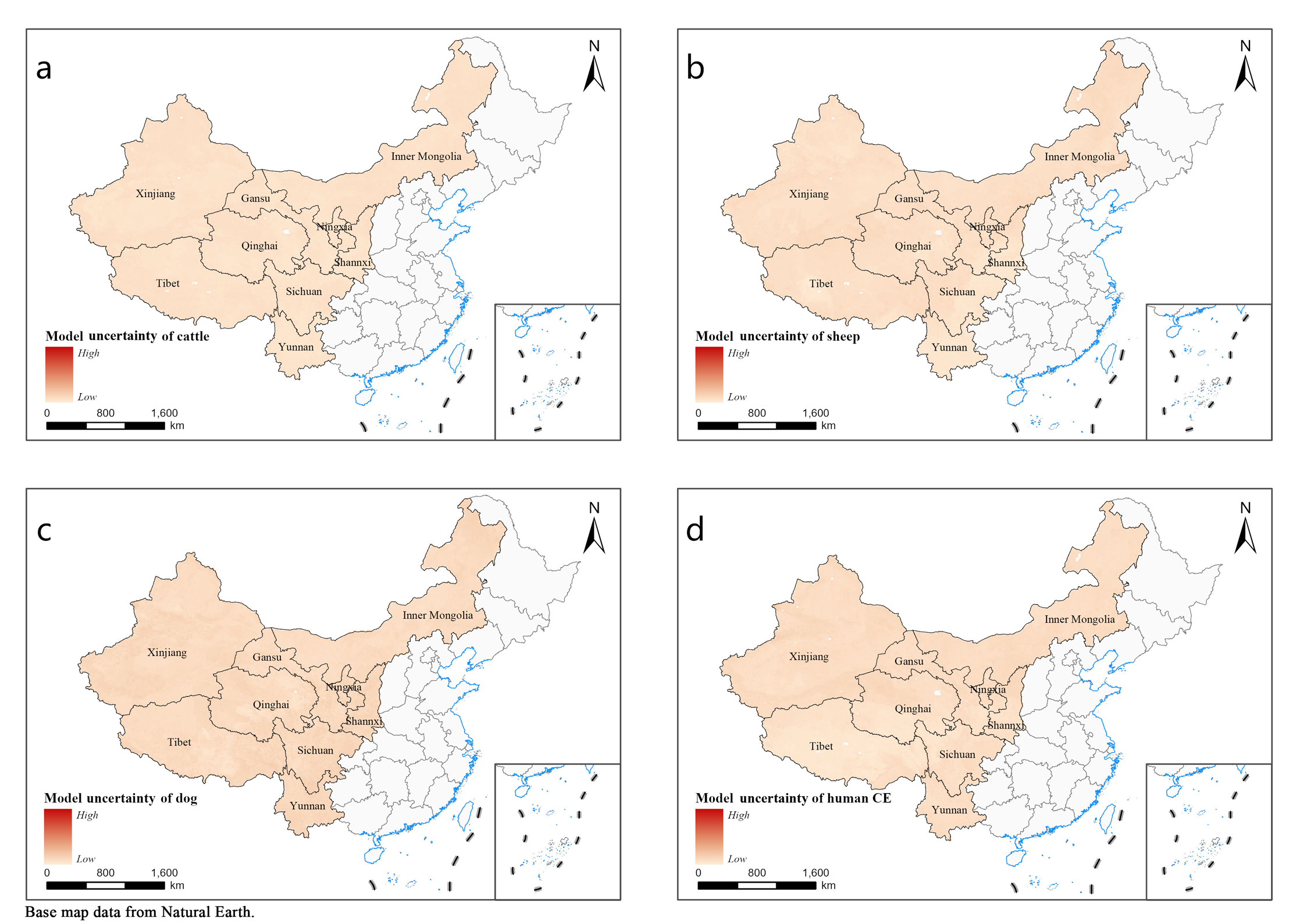

Supplement: S7 Fig — (a) cattle, (b) sheep, (c) dogs, and (d) human CE. Note: Base map data from Map World (https://map.tianditu.gov.cn/). (TIF) [file pntd.0013182.s007.tif]

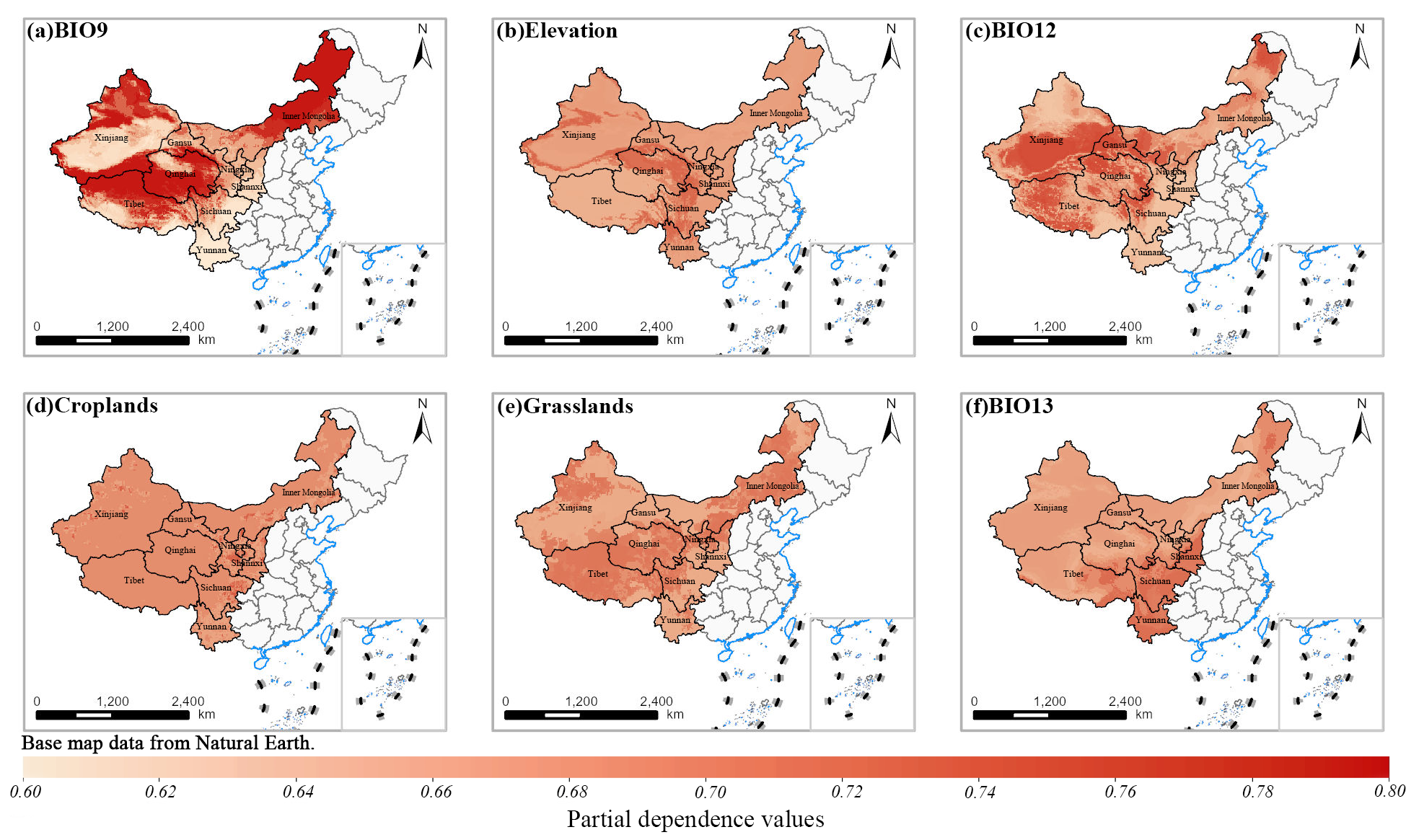

Supplement: S8 Fig — Note: Base map data from Map World (https://map.tianditu.gov.cn/). (TIF) [file pntd.0013182.s008.tif]

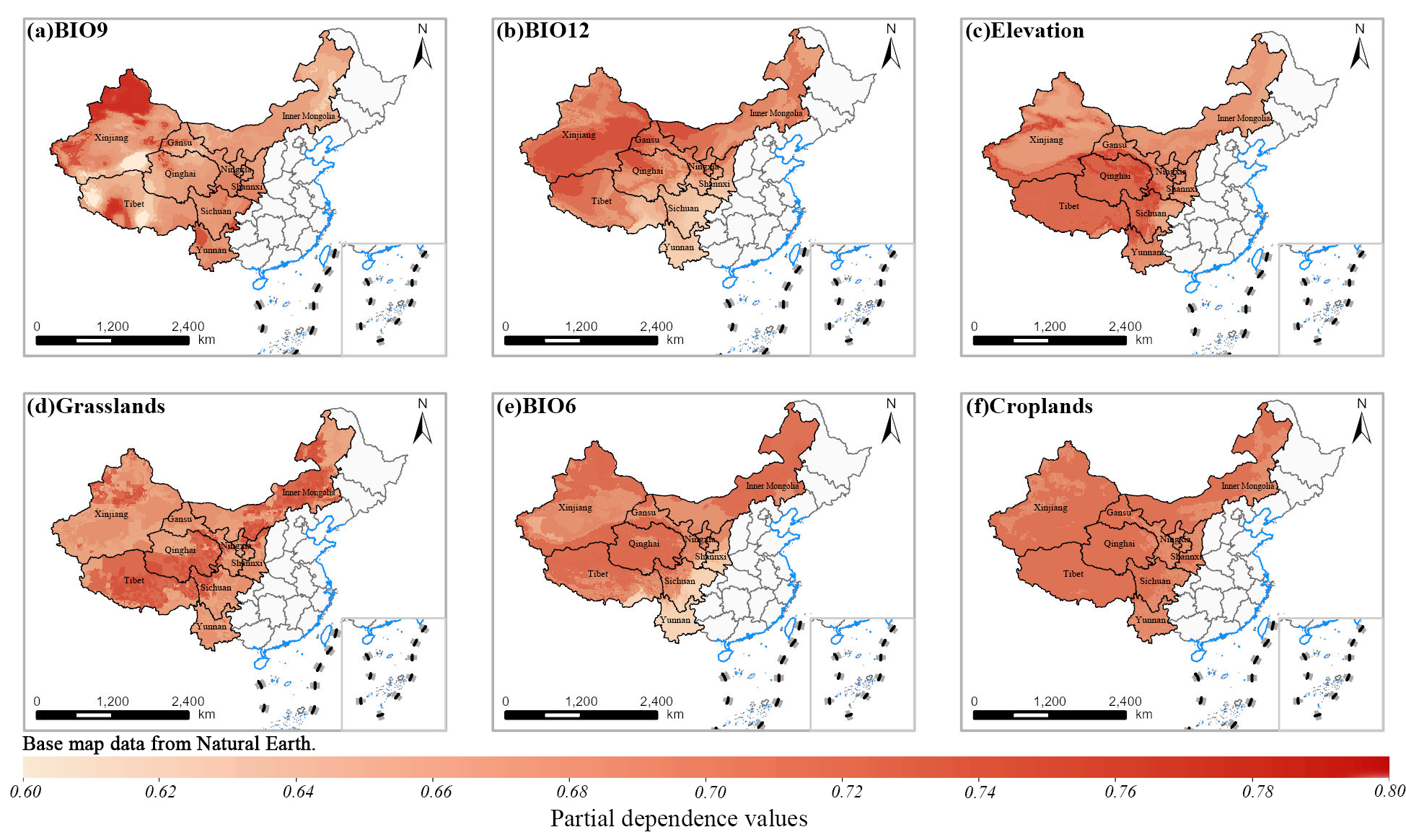

Supplement: S9 Fig — Note: Base map data from Map World (https://map.tianditu.gov.cn/). (TIF) [file pntd.0013182.s009.tif]

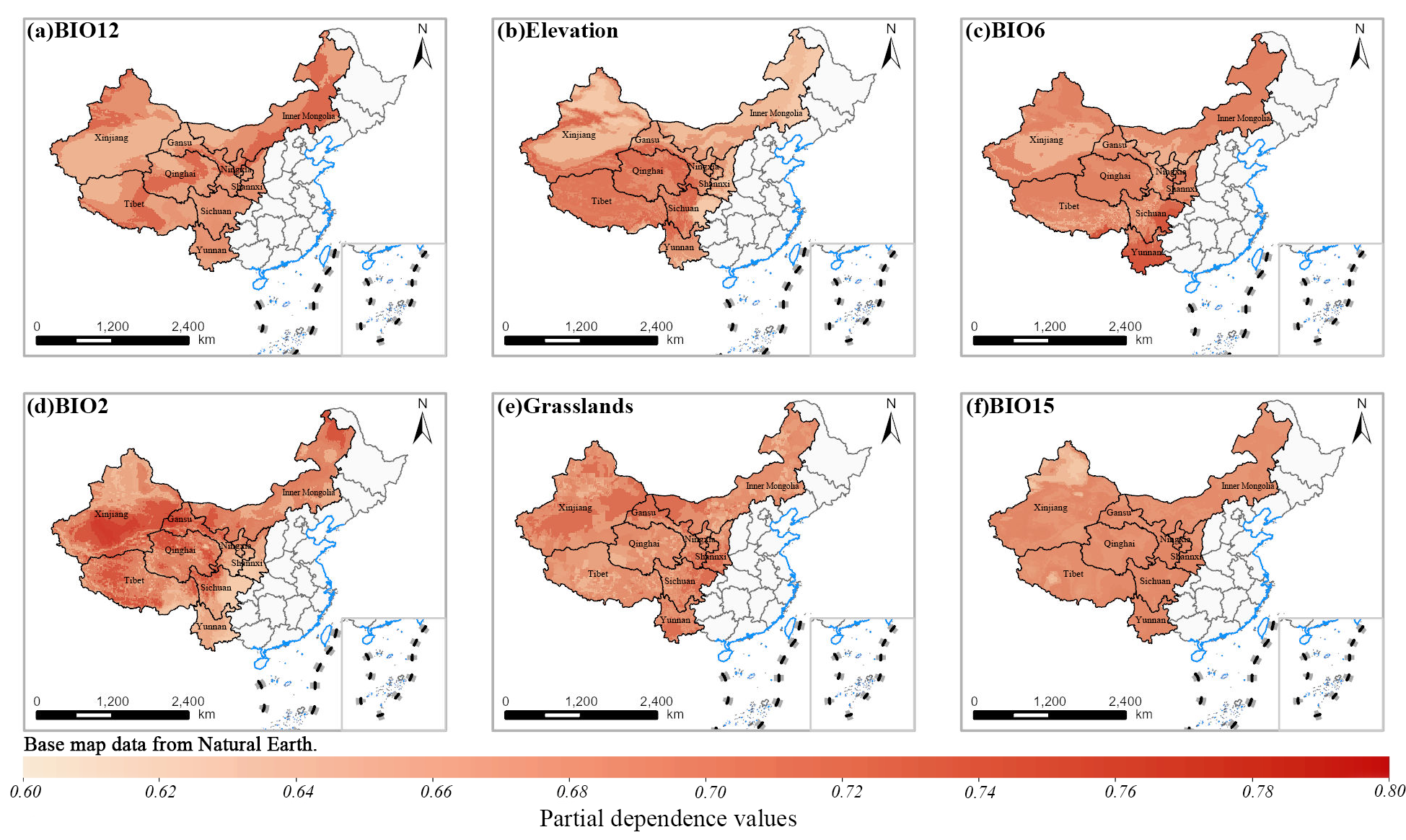

Supplement: S10 Fig — Note: Base map data from Map World (https://map.tianditu.gov.cn/). (TIF) [file pntd.0013182.s010.tif]

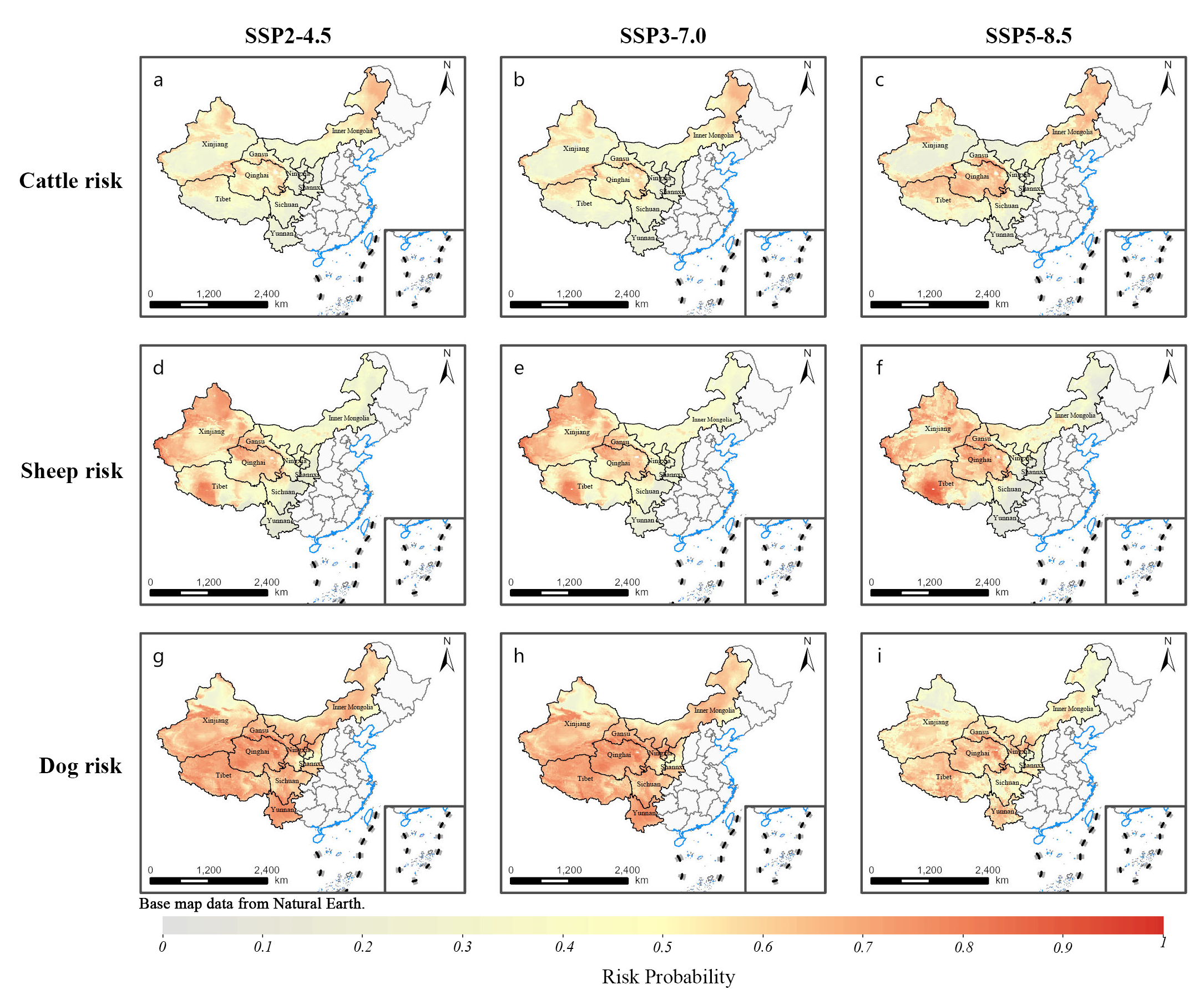

Supplement: S11 Fig — Note: Base map data from Map World (https://map.tianditu.gov.cn/). (TIF) [file pntd.0013182.s011.tif]

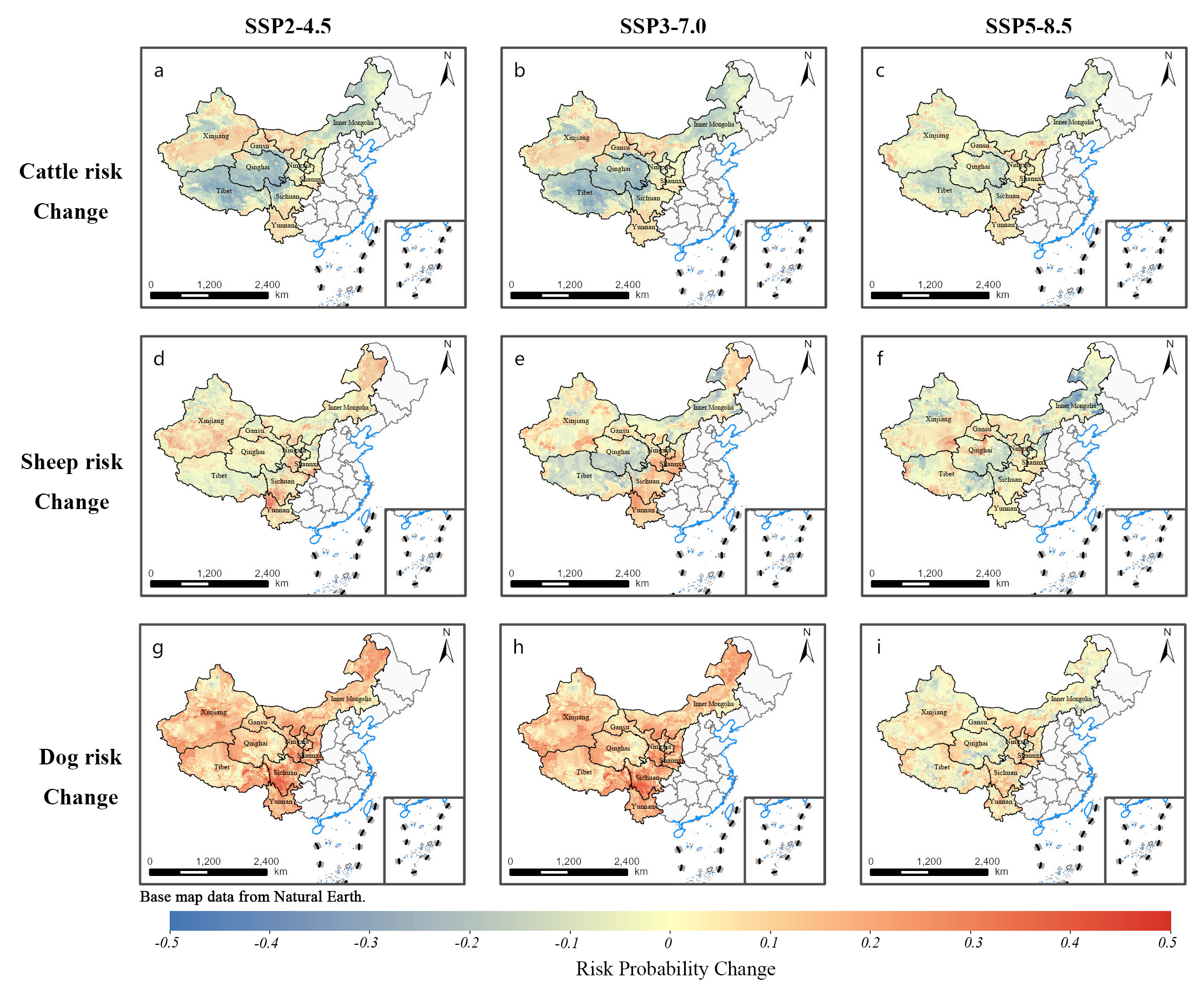

Supplement: S12 Fig — Note: Base map data from Map World (https://map.tianditu.gov.cn/). (TIF) [file pntd.0013182.s012.tif]

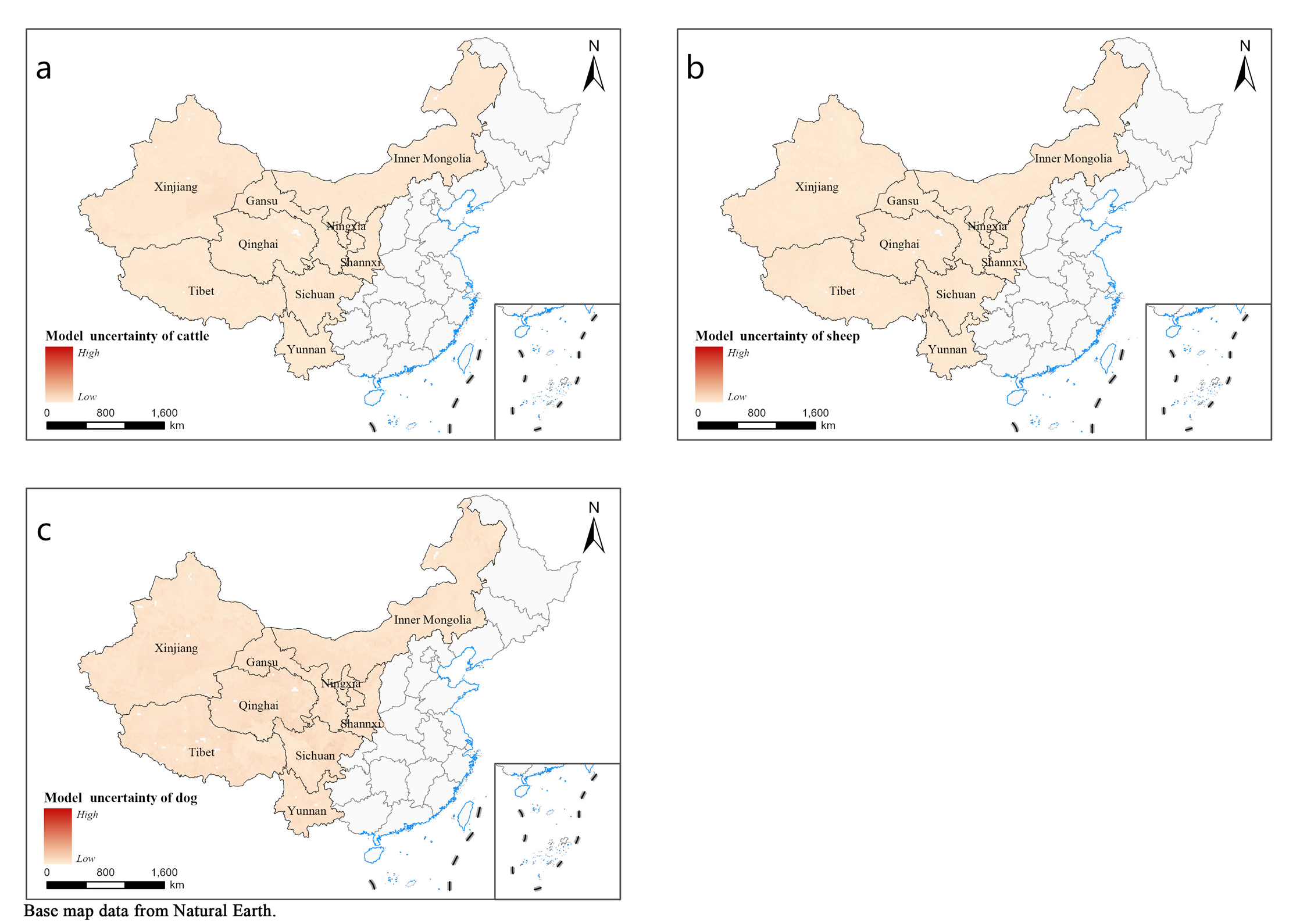

Supplement: S13 Fig — (a) cattle, (b) sheep, (c) dogs. Note: Base map data from Map World (https://map.tianditu.gov.cn/). (TIF) [file pntd.0013182.s013.tif]

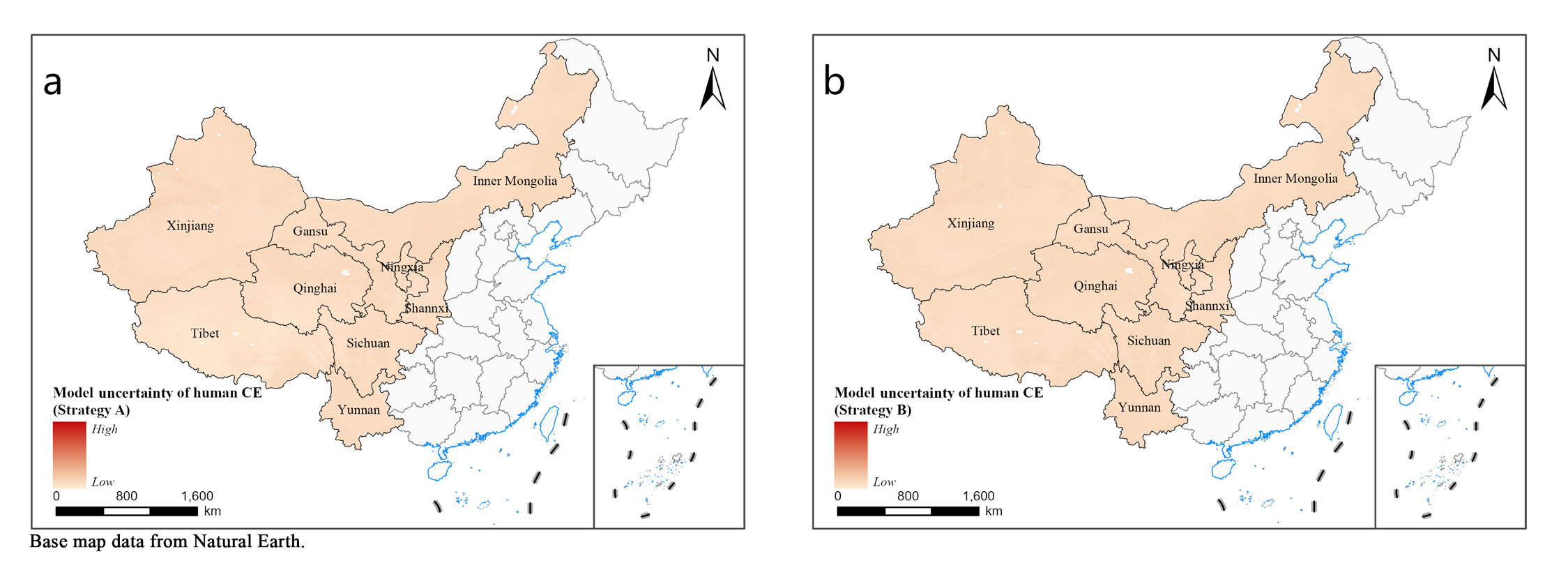

Supplement: S14 Fig — (a) Strategy A, (b) Strategy B. Note: Base map data from Map World (https://map.tianditu.gov.cn/). (TIF) [file pntd.0013182.s014.tif]

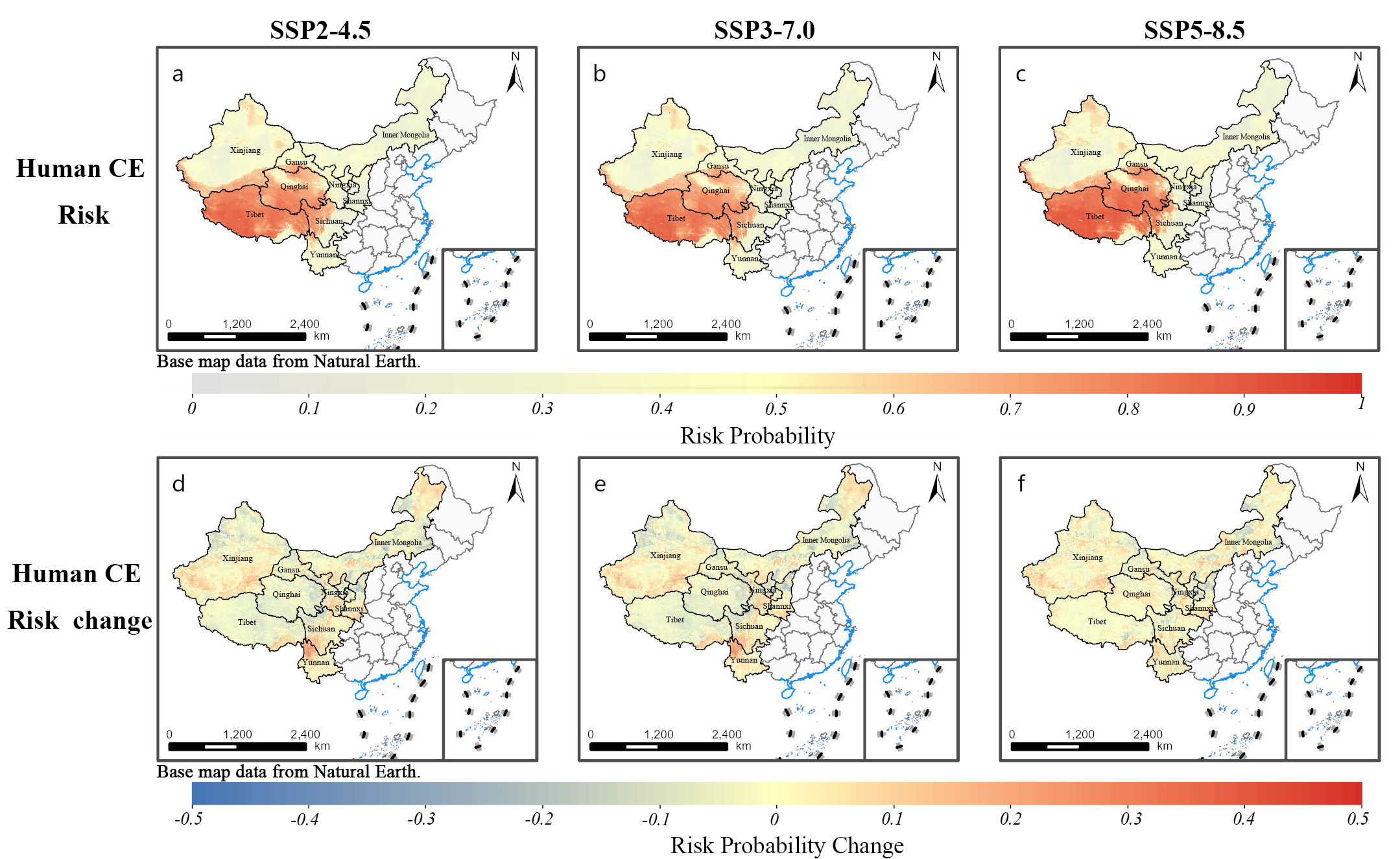

Supplement: S15 Fig — (Strategy A: Awareness Rate Unchanged). Note: Base map data from Map World (https://map.tianditu.gov.cn/). (TIF) [file pntd.0013182.s015.tif]
