# Supplementary material for: Benefits of public awareness in mitigating cystic echinococcosis risk in Western China: A climate and socio-economic perspective
Source: PLoS Negl Trop Dis. 2025 Jul 9;19(7):e0013182. doi: 10.1371/journal.pntd.0013182 (PMC12240338; doi:10.1371/journal.pntd.0013182)
Supplement: S1 Table — (DOCX) [file pntd.0013182.s016.docx]

**S1 Table. Factors potentially associated with the echinococcosis used in the analysis.**

| **Categories** | **Variable** | **Reference** | **Usage and/or result** |
| --- | --- | --- | --- |
| **Ecoclimatic** | **BIO1-19** | Xu J, et al.[1] | BIO12, BIO10, BIO8 were identified as the dominant environmental variables. |
|  |  | Cadavid Restrepo AM, et al.[2] | A positive association between winter mean temperature and a significant nonlinear effect of annual mean temperature with annual CE incidence. |
|  |  | Ma T, et.al[3] | CE prevalence was negatively associated with temperature. |
|  |  | Ma T, et.al[4] | The annual average precipitation was associated with the prevalence of CE at the significance level of P < 0.05. |
| **Geographical** | **Land cover** | Huang D, et.al[5] | The grassland area ratio was an independent variable positively correlated with the prevalence of human CE. |
|  |  | Ma T, et.al[3] | CE prevalence was positively correlated with grass area. |
|  | **Elevation** | Xu J, et al.[1] | Elevation was identified as one of the dominant environmental variables. |
|  |  | Wang L, et al.[6] | The prevalence of human cystic echinococcosis was positively correlated with the average elevation. |
|  |  | Ma T, et.al[3] | CE prevalence was positively correlated with elevation. |
|  |  | Zeng X, et.al[7] | Altitude had a negative association with human CE prevalence. |
|  |  | Ma T, et.al[4] | Elevation was associated with the prevalence of CE at the significance level of P < 0.05. |
| **Socioeconomic** | **Population density** | Huang D, et.al[5] | Used in CE ecological niche model. |
|  |  | Possenti A,et al[8] | Used in CE ecological niche model. |
|  |  | Yin J, et.al[9] | Population was identified as one of the key factors. |
| **Biological** | **Cattle density** | Ma T, et.al[3] | CE prevalence was positively correlated with cattle density. |
|  |  | Ma T, et.al[10] | The cattle population contributes the most for CE. |
|  | **Sheep density** | Zeng X, et.al[7] | Livestock plays the most important role in CE transmission. |

**Supplementary references**

1. Xu J, Song G, Xiong M, Zhang Y, Sanlang B, Long G, et al. Prediction of the potential suitable habitat of Echinococcus granulosus, the pathogen of echinococcosis, in the Tibetan Plateau under future climate scenarios. Environ Sci Pollut Res. 2022;30: 21404–21415. doi:10.1007/s11356-022-23666-6

2. Cadavid Restrepo AM, Yang YR, McManus DP, Gray DJ, Barnes TS, Williams GM, et al. Spatiotemporal patterns and environmental drivers of human echinococcoses over a twenty-year period in Ningxia Hui Autonomous Region, China. Parasit Vectors. 2018;11: 108. doi:10.1186/s13071-018-2693-z

3. Ma T, Wang Q, Hao M, Xue C, Wang X, Han S, et al. Epidemiological characteristics and risk factors for cystic and alveolar echinococcosis in China: an analysis of a national population-based field survey. Parasit Vectors. 2023;16: 181. doi:10.1186/s13071-023-05788-z

4. Ma T, Jiang D, Quzhen G, Xue C, Han S, Wu W, et al. Factors influencing the spatial distribution of cystic echinococcosis in Tibet, China. Sci Total Environ. 2021;754: 142229. doi:10.1016/j.scitotenv.2020.142229

5. Huang D, Li R, Qiu J, Sun X, Yuan R, Shi Y, et al. Geographical Environment Factors and Risk Mapping of Human Cystic Echinococcosis in Western China. Int J Environ Res Public Health. 2018;15: 1729. doi:10.3390/ijerph15081729

6. Wang L, Wang Z, Qin M, Lei J, Cheng X, Yan J, et al. A regressive analysis of the main environmental risk factors of human echinococcosis in 370 counties in China. Periago MV, editor. PLoS Negl Trop Dis. 2024;18: e0012131. doi:10.1371/journal.pntd.0012131

7. Zeng X, Guan Y, Wu W, Wang L, Cai H, Fang Q, et al. Analysis of Factors Influencing Cystic Echinococcosis in Northwest Non-Qinghai Tibetan Plateau Regions of China. Am J Trop Med Hyg. 2020;102: 567–573. doi:10.4269/ajtmh.18-0703

8. Possenti A, Manzano-Román R, Sánchez-Ovejero C, Boufana B, La Torre G, Siles-Lucas M, et al. Potential Risk Factors Associated with Human Cystic Echinococcosis: Systematic Review and Meta-analysis. PLoS Negl Trop Dis. 2016;10: e0005114. doi:10.1371/journal.pntd.0005114

9. Yin J, Wu X, Li C, Han J, Xiang H. The Impact of Environmental and Host Factors on Human Cystic Echinococcosis: A County‐Level Modeling Study in Western China. GeoHealth. 2023;7: e2022GH000721. doi:10.1029/2022GH000721

10. Ma T, Jiang D, Hao M, Fan P, Zhang S, Quzhen G, et al. Geographical Detector-based influence factors analysis for Echinococcosis prevalence in Tibet, China. Grenouillet F, editor. PLoS Negl Trop Dis. 2021;15: e0009547. doi:10.1371/journal.pntd.0009547
