# Supplementary material for: Benefits of public awareness in mitigating cystic echinococcosis risk in Western China: A climate and socio-economic perspective
Source: PLoS Negl Trop Dis. 2025 Jul 9;19(7):e0013182. doi: 10.1371/journal.pntd.0013182 (PMC12240338; doi:10.1371/journal.pntd.0013182)
Supplement: S2 Table — (DOCX) [file pntd.0013182.s017.docx]

**S2 Table. The spatial resolution, study duration, and source of the included data.**

| **Categories** | **Variable** | **Study duration** | **Spatial resolution** | **Source of data** | **Website** |
| --- | --- | --- | --- | --- | --- |
| **Cases** | **Occurrence data** | 2012-2016 | Township level | China CDC | - |
| **Ecoclimatic** | **BIO1-19** | 2007-2016 | 1km | WorldClim | https://www.worldclim.org/ |
|  |  | 2021-2040 | 1km |  |  |
| **Geographical** | **Land cover** | 2007-2016 | 500m | NASA (MCD12Q1) | https://lpdaac.usgs.gov/products/mcd12q1v006/ |
|  |  | 2030 | 1km | figshare | https://figshare.com/articles/dataset/23542860 |
|  | **Elevation** | 2010 | 1km | EarthEnv (DEM90) | http://www.earthenv.org/ |
| **Socioeconomic** | **Population density** | 2007-2016 | 1km | WorldPop | https://www.worldpop.org/ |
|  |  | 2021-2040 |  | NASA(SEDAC) | https://sedac.ciesin.columbia.edu/ |
|  | **Awareness rate** | 2007-2016 | City level | China CDC | - |
| **Biological** | **Cattle density** | 2015 | 10km | Scientific Data | https://www.fao.org/livestock-systems/global-distributions/zh/ |
|  | **Sheep density** | 2015 | 10km |  |  |
