# Supplementary material for: Benefits of public awareness in mitigating cystic echinococcosis risk in Western China: A climate and socio-economic perspective
Source: PLoS Negl Trop Dis. 2025 Jul 9;19(7):e0013182. doi: 10.1371/journal.pntd.0013182 (PMC12240338; doi:10.1371/journal.pntd.0013182)
Supplement: S3 Table — (DOCX) [file pntd.0013182.s018.docx]

**S3 Table. VIF assessment for variables in different models.**

| **VIF value** | **Cattle Infection Risk Model** | **Sheep Infection Risk Model** | **Dog Infection Risk Model** | **Human CE Risk Model (Excluding Awareness)** | **Human CE Risk Model (Including Awareness)** |
| --- | --- | --- | --- | --- | --- |
| BIO2 | 2.00 | 2.00 | 2.01 | 2.89 | 2.91 |
| BIO6 | **38.18** | 1.67 | **21.14** | **26.78** | **26.66** |
| BIO8 | **21.85** | **11.24** | **12.36** | **13.08** | **13.45** |
| BIO9 | 2.00 | **12.28** | **15.78** | 3.50 | 3.50 |
| BIO12 | **40.95** | 2.94 | 3.49 | 4.83 | 4.84 |
| BIO13 | 3.41 | **38.40** | **52.05** | **58.28** | **59.61** |
| BIO14 | **29.65** | **38.47** | **37.75** | 3.96 | 3.97 |
| BIO15 | 2.80 | 2.53 | 2.38 | 5.21 | 5.32 |
| BIO19 | 2.16 | 2.99 | 3.05 | 3.00 | 3.23 |
| Elevation | 4.22 | 2.00 | 2.58 | 3.94 | 4.85 |
| Population density | - | - | 1.42 | 1.41 | 1.41 |
| Forest | 7.95 | 1.96 | 2.47 | 4.50 | 4.56 |
| Grassland | 8.31 | 2.52 | 1.58 | 3.96 | 3.96 |
| Cropland | 3.79 | 2.33 | 2.17 | 3.00 | 3.00 |
| Urban | **16.60** | 1.13 | 1.50 | 1.61 | 1.61 |
| Water | 1.60 | 1.13 | 1.08 | **11.77** | **11.79** |
| Barren | 1.14 | 1.14 | 2.06 | **12.94** | **12.94** |
| Cattle density | 1.35 | - | - | - | - |
| Sheep density | - | - | - | - | - |
| Cattle infection risk | - | - | - | 4.31 | 4.37 |
| Sheep infection risk | - | - | - | 4.60 | 4.72 |
| Dog infection risk | - | - | - | 1.90 | 1.91 |
| Awareness rate | - | - | - | 2.89 | 2.39 |
