# Supplementary material for: Benefits of public awareness in mitigating cystic echinococcosis risk in Western China: A climate and socio-economic perspective
Source: PLoS Negl Trop Dis. 2025 Jul 9;19(7):e0013182. doi: 10.1371/journal.pntd.0013182 (PMC12240338; doi:10.1371/journal.pntd.0013182)
Supplement: S4 Table — (DOCX) [file pntd.0013182.s019.docx]

**S4 Table. Detail information of variables used for modelling in CE infection risk.**

| **Classification of variables** | **Variable** | **Description** | **Cattle infection risk modelling** | **Sheep infection risk modelling** | **Dog infection risk modelling** | **Human CE infection risk modelling**  **(excluding awareness rate)** | **Human CE infection risk modelling**  **(including awareness rate)** |
| --- | --- | --- | --- | --- | --- | --- | --- |
| **Ecoclimatic variables** | **BIO1** | Annual mean temperature (℃) |  |  |  |  |  |
|  | **BIO2** | Mean diurnal range (Mean of monthly (max temp-min temp)) (℃) | √ | √ | √ | √ | √ |
|  | **BIO3** | Isothermality (BIO2/ BIO7) (*100) |  |  |  |  |  |
|  | **BIO4** | Temperature seasonality (standard deviation*100) |  |  |  |  |  |
|  | **BIO5** | Max temperature of warmest month (℃) |  |  |  |  |  |
|  | **BIO6** | Min temperature of coldest month (℃) |  | √ |  |  |  |
|  | **BIO7** | Annual range of temperature (BIO5- BIO6) (℃) |  |  |  |  |  |
|  | **BIO8** | Mean temperature of wettest quarter (℃) |  |  |  |  |  |
|  | **BIO9** | Mean temperature of driest quarter (℃) | √ |  |  | √ | √ |
|  | **BIO10** | Mean temperature of warmest quarter (℃) |  |  |  |  |  |
|  | **BIO11** | Mean temperature of coldest quarter (℃) |  |  |  |  |  |
|  | **BIO12** | Annual precipitation (mm) |  | √ | √ | √ | √ |
|  | **BIO13** | Precipitation of wettest month (mm) | √ |  |  |  |  |
|  | **BIO14** | Precipitation of driest month (mm) |  |  |  | √ | √ |
|  | **BIO15** | Precipitation seasonality (Coefficient of variation) | √ | √ | √ | √ | √ |
|  | **BIO16** | Precipitation of wettest quarter (mm) |  |  |  |  |  |
|  | **BIO17** | Precipitation of driest quarter (mm) |  |  |  |  |  |
|  | **BIO18** | Precipitation of warmest quarter (mm) |  |  |  |  |  |
|  | **BIO19** | Precipitation of coldest quarter (mm) | √ | √ | √ | √ | √ |
| **Geographical variables** | **Elevation** | Average elevation (m) | √ | √ | √ | √ | √ |
|  | **Forest** | Percentage coverage of forests (%) | √ | √ | √ | √ | √ |
|  | **Grasslands** | Percentage coverage of grasslands (%) | √ | √ | √ | √ | √ |
|  | **Croplands** | Percentage coverage of croplands (%) | √ | √ | √ | √ | √ |
|  | **Urban** | Percentage coverage of urban and built-up lands (%) |  | √ | √ | √ | √ |
|  | **Barren** | Percentage coverage of barren (%) | √ | √ | √ |  |  |
|  | **Water** | Percentage coverage of water bodies (%) | √ | √ | √ |  |  |
| **Social-economic variables** | **Cattle density** | The average number of cattle | √ |  |  |  |  |
|  | **Sheep density** | The average number of sheep and goat |  | √ |  |  |  |
|  | **Population density** | Average population density |  |  | √ | √ | √ |
|  | **Awareness rate** | Awareness rate of echinococcosis-related knowledge (%) |  |  |  |  | √ |
| **Biological variables** | **Cattle infection risk** | Average probability of echinococcosis infection in cattle |  |  |  | √ | √ |
|  | **Sheep infection risk** | Average probability of echinococcosis infection in sheep |  |  |  | √ | √ |
|  | **Dog infection risk** | Average probability of echinococcosis infection in dogs |  |  |  | √ | √ |
