# Supplementary material for: Benefits of public awareness in mitigating cystic echinococcosis risk in Western China: A climate and socio-economic perspective
Source: PLoS Negl Trop Dis. 2025 Jul 9;19(7):e0013182. doi: 10.1371/journal.pntd.0013182 (PMC12240338; doi:10.1371/journal.pntd.0013182)
Supplement: S5 Table — (DOCX) [file pntd.0013182.s020.docx]

**S5 Table. Predicted future population proportion and changes in the provinces of western China residing in high-risk areas compared to the 2010s (%).**

| **Province** | **Population proportion (changes) residing in high-risk areas (%)** | | | | | |
| --- | --- | --- | --- | --- | --- | --- |
|  | **Strategy A** | | | **Strategy B** | | |
|  | **SSP2-4.5** | **SSP3-7.0** | **SSP5-8.5** | **SSP2-4.5** | **SSP3-7.0** | **SSP5-8.5** |
| **Inner Mongolia** | 1.62(-3.29) | 0.62(-4.29) | 4.44(-0.47) | 0.00(-4.90) | 0.00(-4.90) | 0.00(-4.90) |
| **Xinjiang** | 14.39(-0.56) | 14.45(-0.50) | 23.37(8.43) | 3.66(-11.28) | 4.07(-10.88) | 11.44(-3.51) |
| **Gansu** | 8.89(1.88) | 7.00(0.00) | 10.92(3.91) | 0.70(-6.31) | 0.73(-6.27) | 1.28(-5.72) |
| **Ningxia** | 2.80(-5.91) | 0.95(-7.76) | 12.80(4.09) | 0.00(-8.71) | 0.00(-8.71) | 0.00(-8.71) |
| **Qinghai** | 45.66(-0.43) | 47.69(1.60) | 45.23(-0.86) | 23.83(-22.26) | 25.41(-20.68) | 33.38(-12.71) |
| **Shaanxi** | 0.39(-3.42) | 0.06(-3.75) | 1.29(-2.52) | 0.00(-3.81) | 0.00(-3.81) | 0.00(-3.81) |
| **Tibet** | 63.54(-12.40) | 65.16(-10.77) | 60.58(-15.35) | 60.76(-15.17) | 62.45(-13.48) | 58.13(-17.81) |
| **Sichuan** | 1.62(-0.06) | 1.79(0.11) | 1.05(-0.63) | 1.23(-0.45) | 1.36(-0.32) | 0.79(-0.89) |
| **Yunnan** | 4.31(3.84) | 4.34(3.87) | 0.51(0.04) | 0.84(0.37) | 0.86(0.39) | 0.02(-0.44) |
| **Total** | 5.18(-1.00) | 4.97(-1.21) | 5.86(-0.31) | 2.05(-4.12) | 2.25(-3.93) | 2.51(-3.66) |
