# Supplementary material for: Benefits of public awareness in mitigating cystic echinococcosis risk in Western China: A climate and socio-economic perspective
Source: PLoS Negl Trop Dis. 2025 Jul 9;19(7):e0013182. doi: 10.1371/journal.pntd.0013182 (PMC12240338; doi:10.1371/journal.pntd.0013182)
Supplement: S6 Table — (DOCX) [file pntd.0013182.s021.docx]

**S6 Table Predicted** **population size changes in the provinces of western China residing in high-risk areas compared to the 2010s (Million).**

| **Province** | **Population size changes** **residing** **in high-risk areas (Million)** | | | | | |
| --- | --- | --- | --- | --- | --- | --- |
|  | **Strategy A** | | | **Strategy B** | | |
|  | **SSP2-4.5** | **SSP3-7.0** | **SSP5-8.5** | **SSP2-4.5** | **SSP3-7.0** | **SSP5-8.5** |
| **Inner Mongolia** | -0.81 | -1.07 | -0.11 | -1.24 | -1.24 | -1.24 |
| **Xinjiang** | -0.61 | -0.51 | 0.94 | -2.65 | -2.55 | -1.25 |
| **Gansu** | 0.49 | 0.07 | 0.91 | -1.62 | -1.6 | -1.48 |
| **Ningxia** | -0.39 | -0.50 | 0.19 | -0.56 | -0.56 | -0.56 |
| **Qinghai** | -0.48 | -0.28 | -0.61 | -1.52 | -1.39 | -1.15 |
| **Shaanxi** | -1.29 | -1.41 | -0.96 | -1.43 | -1.43 | -1.43 |
| **Tibet** | -1.33 | -1.07 | -1.66 | -1.41 | -1.15 | -1.72 |
| **Sichuan** | 0.02 | 0.22 | -0.50 | -0.31 | -0.15 | -0.71 |
| **Yunnan** | 1.49 | 1.60 | -0.03 | 0.11 | 0.14 | -0.21 |
| **Total** | -2.92 | -2.95 | -1.82 | -10.63 | -9.93 | -9.74 |
